# Chitosan-stabilized electrospun nanofiber dressings with embedded flame-made nanosilver for the treatment of resistant infections

**DOI:** 10.1016/j.isci.2026.117991

**Published:** 2026-10-23

**Authors:** Reshma V. Ramachandran, Jennifer Geara Semaan, Maria Samara, Thomas Thersleff, Matthew Fielden, Pehr Sommar, Ning Xu Landén, Georgios A. Sotiriou

**Affiliations:** 1 Department of Microbiology, Tumor and Cell Biology, Karolinska Institutet, Stockholm, Sweden; 2 Department of Chemistry, Stockholm University, Sweden; 3 Science For Life Laboratory (SciLifeLab), Stockholm University, Solna, Sweden; 4 Dermatology and Venereology Division, Department of Medicine Solna, Center for Molecular Medicine, Karolinska Institutet, 17176 Stockholm, Sweden; 5 Department of Cell and Molecular Biology, Karolinska Institutet, Stockholm, Sweden; 6 School of Chemistry, Biotechnology and Health, KTH Royal Institute of Technology, Stockholm, Sweden; 7 Department of Plastic and Reconstructive Surgery, Karolinska University Hospital, Stockholm, Sweden; 8 Department of Molecular Medicine and Surgery, Karolinska Institutet, Stockholm, Sweden

**Keywords:** wound dressing, flame spray pyrolysis, Ag, nanosilver, antibacterial, electrospinning, skin infections

## Abstract

Wound healing can be severely compromised by infections, particularly those caused by antibiotic-resistant bacteria such as methicillin-resistant *Staphylococcus aureus* (MRSA) and *Pseudomonas aeruginosa*. We developed antibacterial wound dressings based on electrospun polyvinyl alcohol-chitosan nanofibers containing flame-made silver nanoparticles (Ag NPs). The 2–10 nm Ag NPs are embedded within ∼200 nm fibers, enabling a high and sustained Ag^+^ ion release with no detectable nanoparticle release, which may help mitigate concerns associated with nanoparticle exposure. Chitosan structurally stabilizes the fibers while modulating swelling and local pH to enhance Ag^+^ diffusion. The dressings achieved >99.9999% bacterial reduction *in vitro* and up to 99% *ex vivo* using freshly excised human skin. Crucially, the dressings maintained >90% viability of human keratinocytes and fibroblasts and showed no detectable impairment of short-term wound healing. This study introduces a scalable, safe-by-design nanofiber platform for infection-resistant and healing-compatible wound care and supports its further evaluation in preclinical studies.

## Highlights

• Flame-made Ag/SiO_2_ nanoparticles enable safe-by-design wound dressings
• Electrospun PVA-chitosan fibers sustain Ag^+^ release without nanoparticle leaching
• Low silver loading eradicates wound pathogens while maintaining cytocompatibility
• *Ex vivo* human skin validates antibacterial efficacy and wound-healing compatibility

## Graphical abstract

**Figure:**
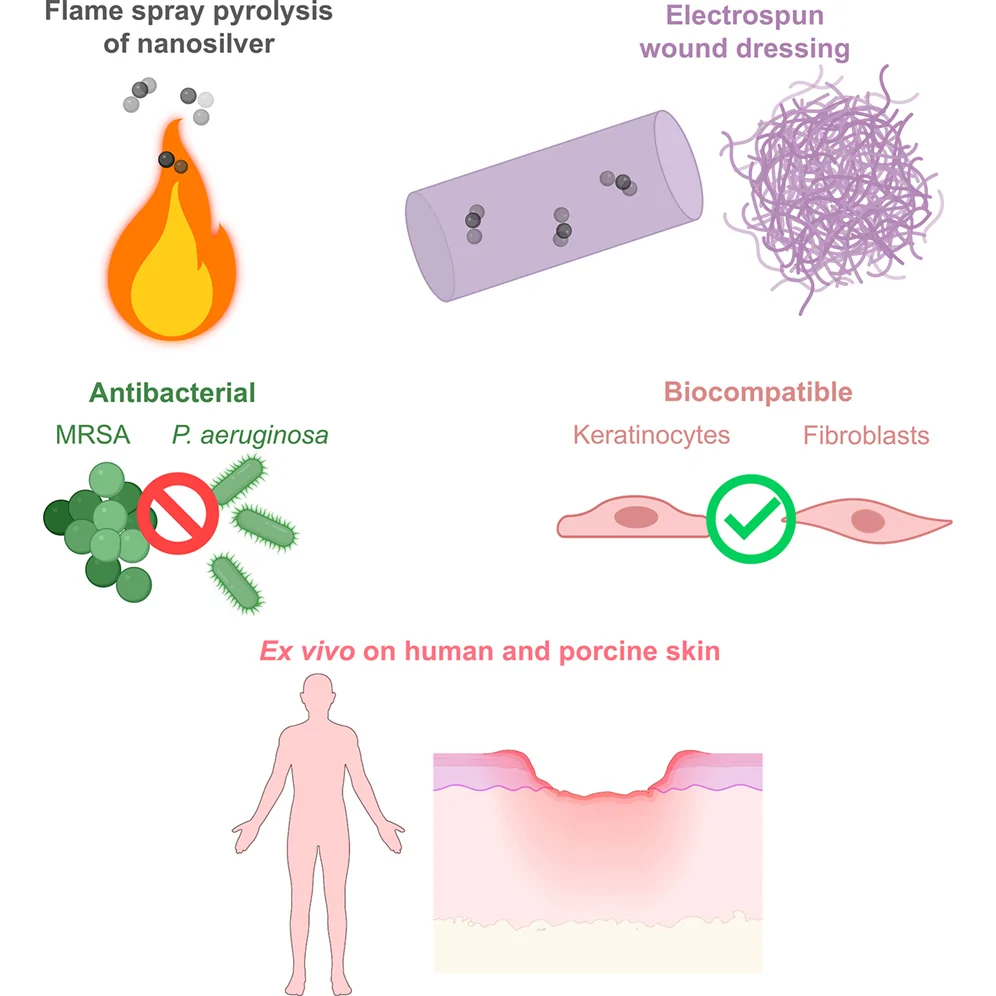

## Teaser

Bioengineering; Tissue engineering; Biotechnology; Materials science; Biomaterials

## Introduction

Wound healing is a complex biological process that involves tissue repair and the restoration of functionality.^1^^,^^2^ The process can be significantly hindered by wound infections, which arise when microorganisms penetrate the wound due to a breach in the skin barrier and local microbial colonization.^3^ Acute wound infections are particularly prevalent in burns, surgical sites, and traumatic wounds, with the primary pathogens being gram-positive methicillin-resistant *Staphylococcus aureus* (MRSA) and gram-negative *Pseudomonas aeruginosa* (*P. aeruginosa*).^4^ These infections can lead to serious health complications, including delayed healing, septicemia, and deep tissue and bone infections, resulting in decreased mobility, financial strain, and potential limb amputation.

Antibiotics such as sulfonamides, penicillin, streptomycin, tetracycline, and vancomycin have been extensively used to treat infections through intravenous, oral, or topical administration.^3^ However, their widespread use and misuse have led to a global antibiotic resistance crisis, with approximately 70% of bacteria causing wound infections becoming resistant to at least one antibiotic, resulting in over 700,000 deaths annually.^5^ Surgical debridement, which involves the removal of necrotic infected tissue, is another treatment approach, but it carries a significant risk of reinfection.^6^ An alternative is the use of antimicrobial wound dressings to prevent or treat wound infections.^7^ These dressings may contain antiseptics such as iodine (e.g., Iodosorb) and chlorhexidine acetate (e.g., Bactigras), or ionic (e.g., Aquacel AG) and nano-crystalline silver (e.g., ACTICOAT).^8^^,^^9^

Silver possesses multiple complex antibacterial mechanisms that reduce the likelihood of bacterial resistance development and enhance its efficacy against existing antibiotic-resistant strains and biofilms.^10^ Despite these advantages, the widespread application of silver-based dressings has been hindered by concerns regarding toxicity,^11^^,^^12^ inconsistent evidence of efficacy, and cost-effectiveness.^13^^,^^14^ Silver nanoparticles (Ag NPs) with dimensions of less than 10 nm, due to their small size and high surface energy, facilitate a sustained high release of Ag^+^ ions, thereby providing significant antibacterial activity.^15^^,^^16^^,^^17^ However, issues related to the accumulation and long-term toxicity of these nanoparticles (NPs) persist.^18^ To ensure the effective deployment of silver-based dressings, it is imperative to utilize a production method that ensures a sufficiently high release of Ag^+^ ions while minimizing nanoparticle release and maintaining biocompatibility.^14^ This necessitates a scalable and reproducible method that allows precise control over the size and morphology of the NPs while also minimizing product development costs.

In this study, we tackle the challenge of developing effective antibacterial wound dressings by employing two industrially relevant manufacturing processes: flame spray pyrolysis (FSP) and electrospinning, as summarized in Figure 1. Antimicrobial Ag/SiO_2_ NPs are synthesized using FSP and then incorporated into polymer fibers through electrospinning to fabricate a multi-scale nanofibrous membrane. The antibacterial efficacy of these membranes is evaluated against MRSA and *P. aeruginosa* using *in vitro* and *ex vivo* models on porcine and human skin. Finally, we test the biocompatibility of electrospun membranes on human skin to address potential toxicity concerns.

**Figure 1:**
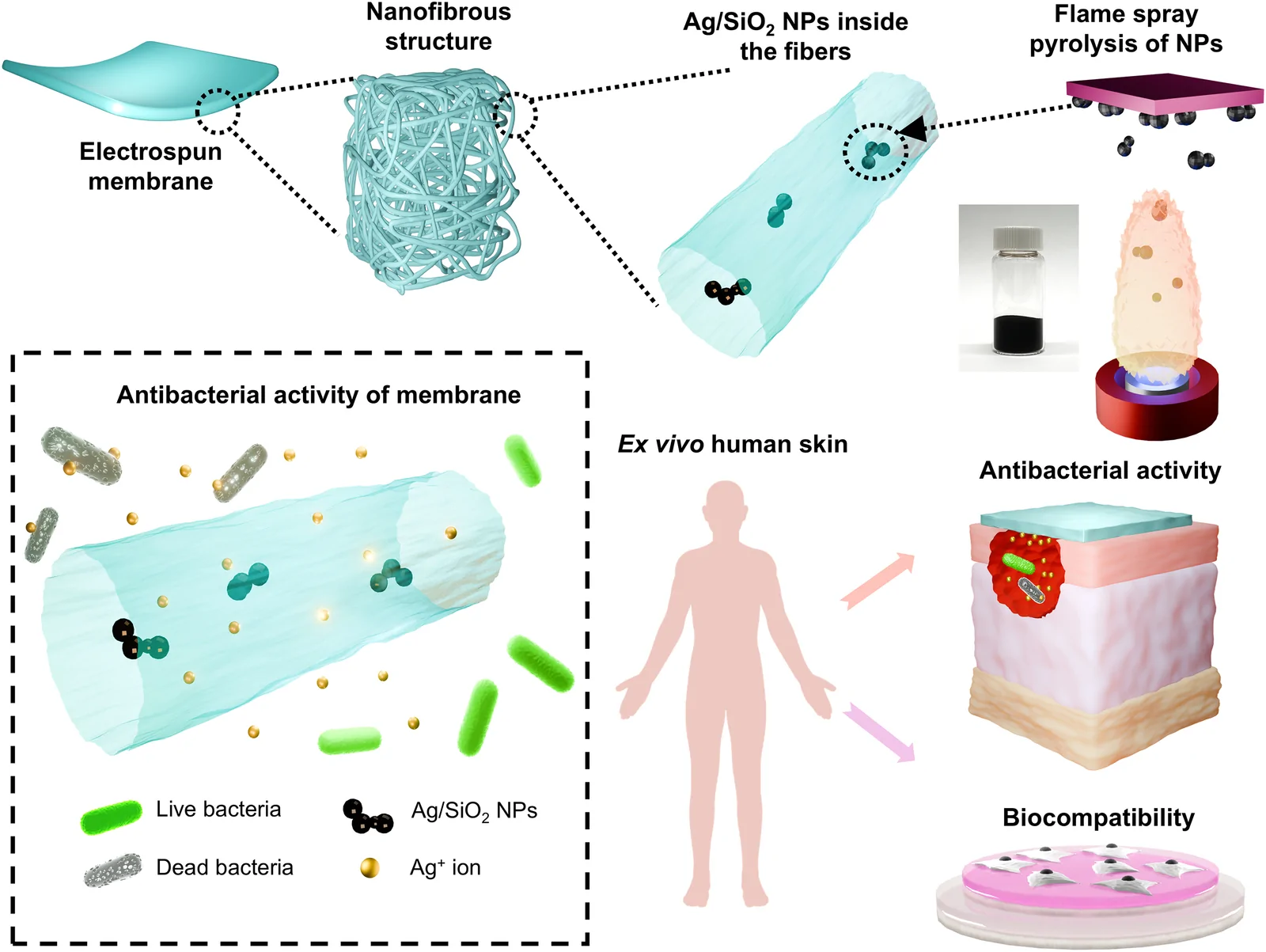
Schematic representation of antibacterial electrospun membranes incorporating flame-made Ag/SiO_2_ nanoparticles The membrane is fabricated through the electrospinning of polyvinyl alcohol and chitosan, resulting in a nanofibrous architecture where each fiber encapsulates Ag/SiO_2_ NPs produced via flame spray pyrolysis. The membrane composition enables a sustained high release of Ag^+^ ions, which exhibit potent activity against MRSA and *P. aeruginosa* infection on human skin. The minimal silver content and the prolonged Ag^+^ ion release, without detectable nanoparticle release, contribute to the membrane’s biocompatibility on human skin.

## Results and discussion

### Scalable fabrication of electrospun membranes

Figure 2A illustrates the fabrication process of electrospun membranes. Multi-component Ag/SiO_2_ (50% w/w) NPs were synthesized using FSP where the amorphous SiO_2_ serves to inhibit the growth of silver during FSP and prevents their agglomeration in aqueous environments.^15^ We had previously demonstrated the antibacterial activity of these particles,^19^ which were then incorporated into a polymer solution prior to electrospinning. The polymers polyvinyl alcohol (PVA) and chitosan (CS) were chosen due to their biocompatibility and suitability for electrospinning.^20^^,^^21^ The solubility of these polymers in aqueous solvents was also beneficial, as these green solvents prevented the precipitation of Ag NPs and provided a conducive environment for the future incorporation of biomolecules. The solvent used here was 2% v/v acetic acid, as CS needs an acidic environment for dissolution. Tween 20 and glycerol were added to the mixture to act as a surfactant and a plasticizer, respectively. Finally, the NP-polymer mixture was electrospun into nanofibers.

**Figure 2:**
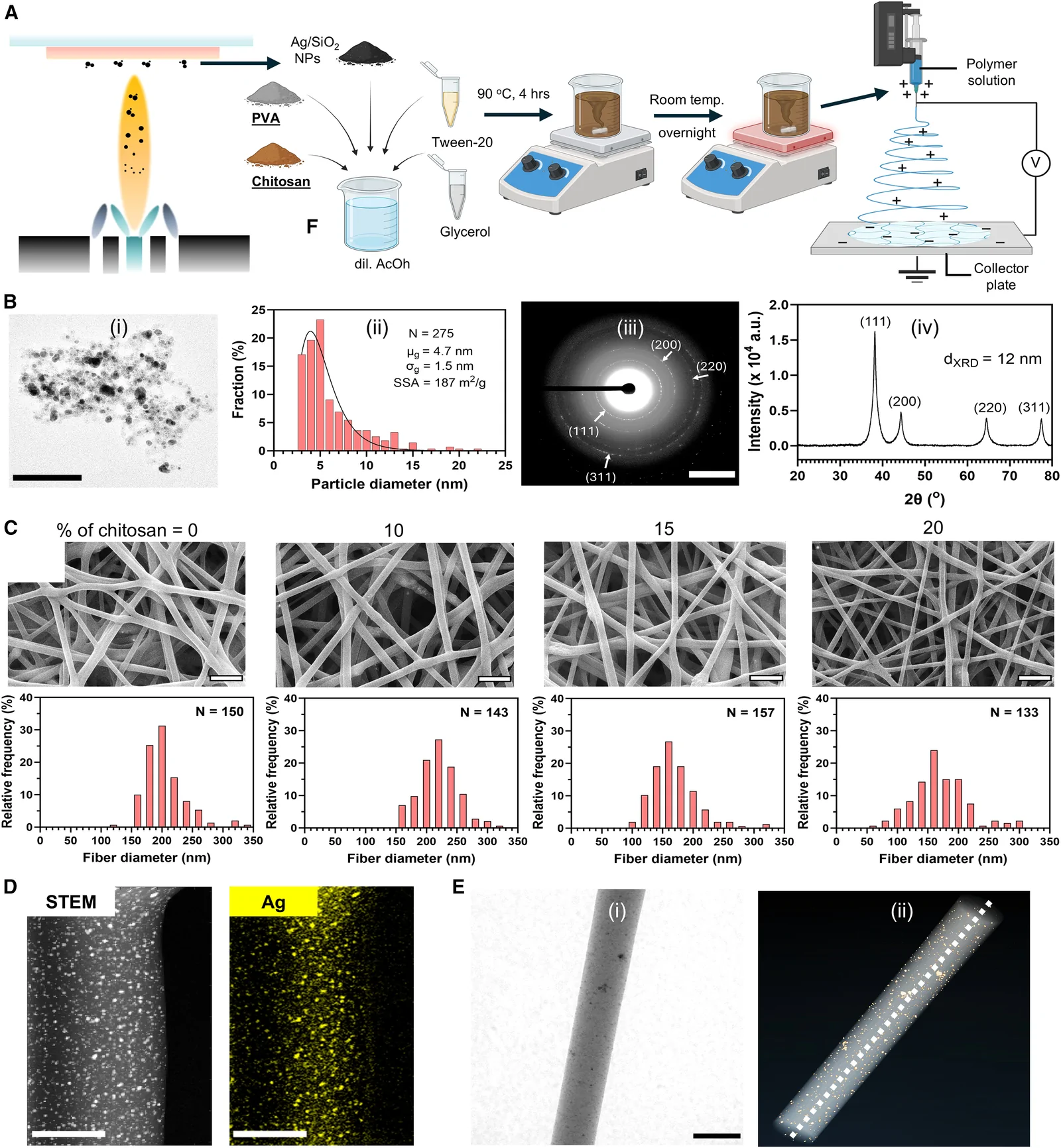
Fabrication and characterization of electrospun membranes (A) Schematic of the fabrication protocol. (B) (i) TEM image of the flame-made Ag/SiO_2_ NPs. Scale bar, 100 nm. (ii) Distribution of the particle diameter. μ_g_ and σ_g_ are calculated from the lognormal fit. Specific surface area (SSA) was obtained from Brunauer-Emmett-Teller analysis. (iii) Selected-area electron diffraction (SAED) pattern of the NPs, with the crystal planes of silver annotated. Scale bar, 5 nm^−1^. (iv) Diffractograms of the NPs showing characteristic peaks of silver. (C) SEM images of the membranes containing Ag/SiO_2_ NPs and the corresponding distribution of fiber diameters. Amount of chitosan (CS) varies as 0, 5, 10, and 20% of the total weight of polymer. Scale bar, 1 μm. (D) HAADF-STEM image and false-color EDX map generated with the Hypermodal Data Fusion method, showing the elemental composition of silver in a single fiber of a membrane containing Ag/SiO_2_ NPs and 20% CS. Scale bar, 100 nm. (E) Electron tomography of a single fiber of a membrane containing Ag/SiO_2_ NPs and 20% CS. (i) Representative 2D BF-TEM image from the tilt series for electron tomography. Scale bar, 100 nm. (ii) Visualization of the 3D model constructed using electron tomography. The fiber axis is shown by a dotted line.

Figure 2Bi shows an electron microscopy image of one Ag/SiO_2_ nanoaggregate in which the dark contrast regions are the Ag NPs that are supported on the amorphous SiO_2_ (gray particles). Figure 2Bii denotes the particle size distribution of the Ag NPs that have a lognormal distribution (σ_g_ = 1.5) with a mean diameter of 4.7 nm. This sub-10 nm average size is critical to enable high Ag^+^ ion release profiles as will be shown later. The particle crystallinity is confirmed by the selected area electron diffraction (SAED) patterns as shown in Figure 2Biii, where the lattice planes correspond to the presence of crystalline silver. The crystallinity of the as-prepared Ag/SiO_2_ NPs is further validated by the X-ray diffraction (XRD) pattern (Figure 2Biv), which exhibits characteristic peaks corresponding to silver metal. The average Ag crystal size as per Rietveld analysis is approximately 12 nm. Rietveld analysis provides a volume-weighted average diameter, which is disproportionately influenced by the presence of a few larger crystalline particles (please see Supplemental Information (SI), Figure S1), while transmission electron microscopy (TEM) analysis yields a number-weighted size distribution.^22^ This discrepancy accounts for the larger diameter observed in XRD compared to TEM analysis.

Figure 2C presents scanning electron microscopy (SEM) images of the electrospun membranes incorporated with Ag/SiO_2_ NPs and the frequency distributions of fiber diameters calculated from these images. The membranes have a nanofibrous structure that ensures a high surface area for the release of antibacterial agents and maintains a moist environment for wound healing. The membranes were fabricated with an increasing amount of CS, 0–20% w/w of the total amount of polymer, while keeping the amount of Ag/SiO_2_ NPs constant. This allows a systematic evaluation of the effect of the membrane composition on the antibacterial activity. As the CS content increases, the viscosity of the polymer solution also rises, requiring a higher voltage for successful electrospinning. This adjustment leads to the formation of thinner fibers. Nevertheless, the fiber diameters for all membranes remain within the range of 150–300 nm with a porosity of around 40%. The miscibility of the polymers PVA and CS is demonstrated by Fourier transform infrared spectroscopy (FTIR) spectra, which show the characteristic peaks of the polymers in all the membranes (see SI, Figure S2). The presence of Ag/SiO_2_ NPs inside the electrospun fibers is further confirmed by the XRD patterns of the fibers that display the characteristic Ag peak around 38° (see SI, Figure S3).

Figure 2D shows the high-angle annular dark-field scanning transmission electron microscopy (HAADF-STEM) image of the Ag/SiO_2_ NPs within a single fiber of an electrospun membrane containing 20% CS. The false-color energy dispersive X-ray spectrometer (EDX) map generated using hypermodal data fusion^23^ confirms the presence of silver. Figure 2Ei shows a 2D bright-field TEM (BF-TEM) image of the fiber from the electron tomography tilt series, while Figure 2Eii and Video S1 display a 3D reconstruction of the fiber from the tomogram. The radial and axial distributions of the Ag/SiO_2_ NPs within the fibers suggest a homogeneous distribution of NPs across various CS concentrations (see SI, Figures S4 and S5).

Video S1. Video showing 3D reconstruction of a single fiber of electrospun membrane (MP4)

### Characterization of membranes and mechanism of Ag^+^ ion release

Upon contact with water, fibers with 0% CS, i.e., 100% PVA, dissolved rapidly, fully releasing the incorporated Ag/SiO_2_ NPs (Figure 3A). Ultraviolet-visible (UV-Vis) spectroscopy (see SI, Figure S6) confirms that Ag/SiO_2_ NPs are released in membranes devoid of CS, while they remain entrapped within membranes containing CS. The supernatant from CS-free membranes exhibits a distinct peak at 420 nm, indicative of the surface plasmon resonance of Ag NPs. However, there was no NP release was detected by UV-Vis spectroscopy under the tested 24 h conditions from the CS-containing fibers suggesting that CS addition prevents the PVA dissolution and traps the Ag/SiO_2_ NPs inside the polymer matrix.

**Figure 3:**
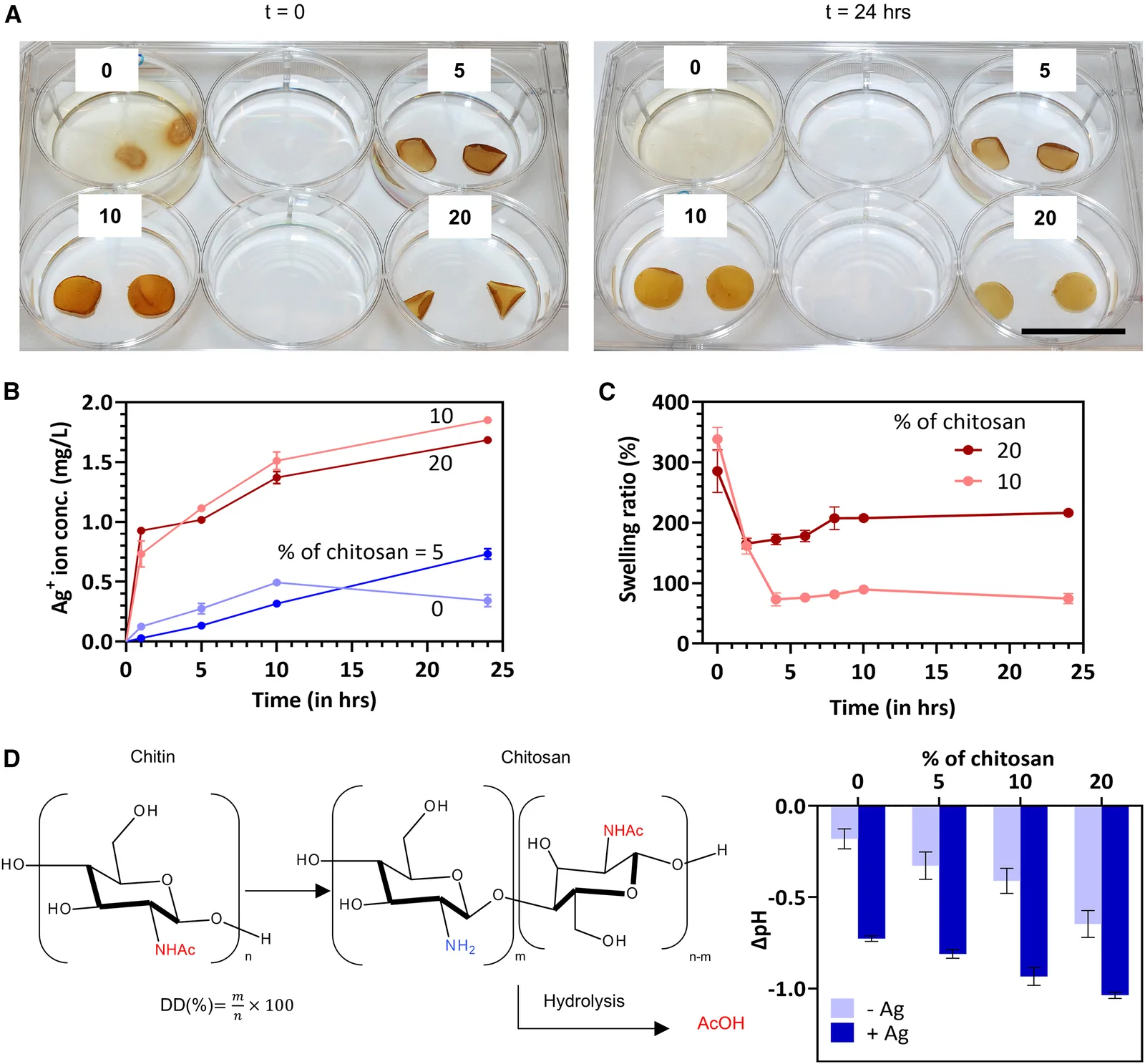
Investigation of Ag^+^ release from electrospun membranes (A) Images showing the dissolution of the membranes having 0%, 5%, 10%, and 20% chitosan in de-ionized water after 0 and 24 h. Scale bar, 2 cm. (B) Release profile of Ag^+^ in de-ionized water at ambient temperature. Data represented as mean ± SD (*N* = 3). (C) Swelling of membranes containing Ag/SiO_2_ NPs and 10% and 20% of chitosan after immersion in deionized water at ambient temperature. Data represented as mean ± SEM (*N* = 3). (D) Change in the pH of deionized water 24 h after immersion of membranes containing Ag/SiO_2_ NPs and various amounts of chitosan. The chemical structure of chitosan is shown which includes a fraction of unhydrolyzed acetyl groups (NHAc). The degree of deacetylation (DD) of chitosan used here is 75%–85%. When the membranes come in contact with an aqueous environment, the resulting hydrolysis releases acetic acid (AcOH), which decreases the pH. Data are represented as mean ± SEM (*N* = 3), where *N* is the number of membrane samples tested.

Figure 3B illustrates the amount of Ag^+^ ions released from the electrospun membranes over a 24-h period. The membranes with 10 and 20% CS exhibit the highest Ag^+^ ion release profiles enabled by the ultra-small sub-10 nm size of the Ag NPs, with no statistically significant difference between 10 and 20% CS. This is particularly interesting as the amount of silver in 1 mg of membrane is just 20 μg as observed from the inductively coupled plasma-optical emission spectroscopy (ICP-OES) measurements (see SI, Figure S7), i.e., a 1 cm × 1 cm membrane contains approximately 20 μg of silver. The high release of silver ions (Ag^+^) from a minimal amount of Ag/SiO_2_ NPs is due to the dissolution of the intrinsic silver oxide layer on the Ag NP surface.^24^^,^^25^ This effect is particularly pronounced in membranes with higher CS concentrations (10% and 20%), indicating the crucial role of the polymer in facilitating Ag^+^ ion release.

To understand better the reasons for the higher Ag^+^ ion release from the membranes containing CS, we performed a detailed physicochemical characterization to study the effects of swelling and local pH upon incubation in aqueous solutions. Figure 3C shows the swelling behavior of electrospun membranes containing 10% and 20% CS in deionized water. Both PVA and CS are hydrophilic polymers that readily absorb water. During this process, free hydroxyl (-OH) and amine (-NH_2_) groups form hydrogen bonds with water molecules, leading to membrane swelling.^26^ In the dry state, membranes have hydrogen bonds within the polymer chains, which break upon initial exposure to water, causing an initial decrease in swelling before full hydration occurs. Substantial swelling creates more space for the diffusion of Ag^+^ ions from Ag/SiO_2_ NPs, contributing to high Ag^+^ ion release.

Figure 3D illustrates the decrease in pH of deionized water following immersion of the membranes showing that the greater the CS content, the more significant the pH reduction. Increased acidity in membranes with higher CS content promotes Ag dissolution, releasing higher concentrations of Ag^+^ ions. This phenomenon can be attributed to the chemical structure of CS, depicted in Figure 3D. CS is derived from the deacetylation of naturally occurring chitin, with a degree of deacetylation ranging from 75% to 85%, implying the presence of residual acetyl groups.^27^ These groups can undergo hydrolysis in an aqueous environment, forming acetic acid and decrease the pH. Silica (SiO_2_) enhances hydrolysis,^28^ which would explain the further reduction in pH for membranes containing Ag/SiO_2_ NPs. Ag^+^ ions are released from Ag through a redox reaction^29^ that can be facilitated by the decrease in pH, thereby increasing Ag^+^ release in the presence of higher CS concentrations. Additionally, as the pH decreases, the amine groups of CS become protonated, leading to increased swelling due to electrostatic repulsion between polymer chains.^30^^,^^31^ This accounts for the higher swelling ratio (200%) observed in membranes with 20% CS in Figure 3C compared to those with 10% CS (100%).

It should be noted that the process of electrospinning that includes high voltages (10–18 kV) does not affect the oxide to metallic silver ratio in the membranes,^32^ as measured using X-ray photoelectron spectroscopy (XPS) (see SI, Figure S8), and thus has no effect on the Ag^+^ ion release profiles of the membranes.

### *In vitro* and *ex vivo* antibacterial activity of the membranes

Next, we evaluated the antibacterial properties of the electrospun membranes against MRSA and *P. aeruginosa*—key pathogens causing wound infections.^4^ The bacteria were incubated with the membranes for 18 h followed by quantification of the colony-forming units (CFUs). Figure 4A displays dot spots of 10-fold dilutions of the bacterial culture alongside the quantification of CFU reduction. Membranes containing 10% and 20% CS exhibited 8-log and 10-log reductions (>99.9999%) in bacterial CFUs, respectively. This observation aligns with the data in Figure 3B, which demonstrated a sustained high release of Ag^+^ ions from these membranes.

**Figure 4:**
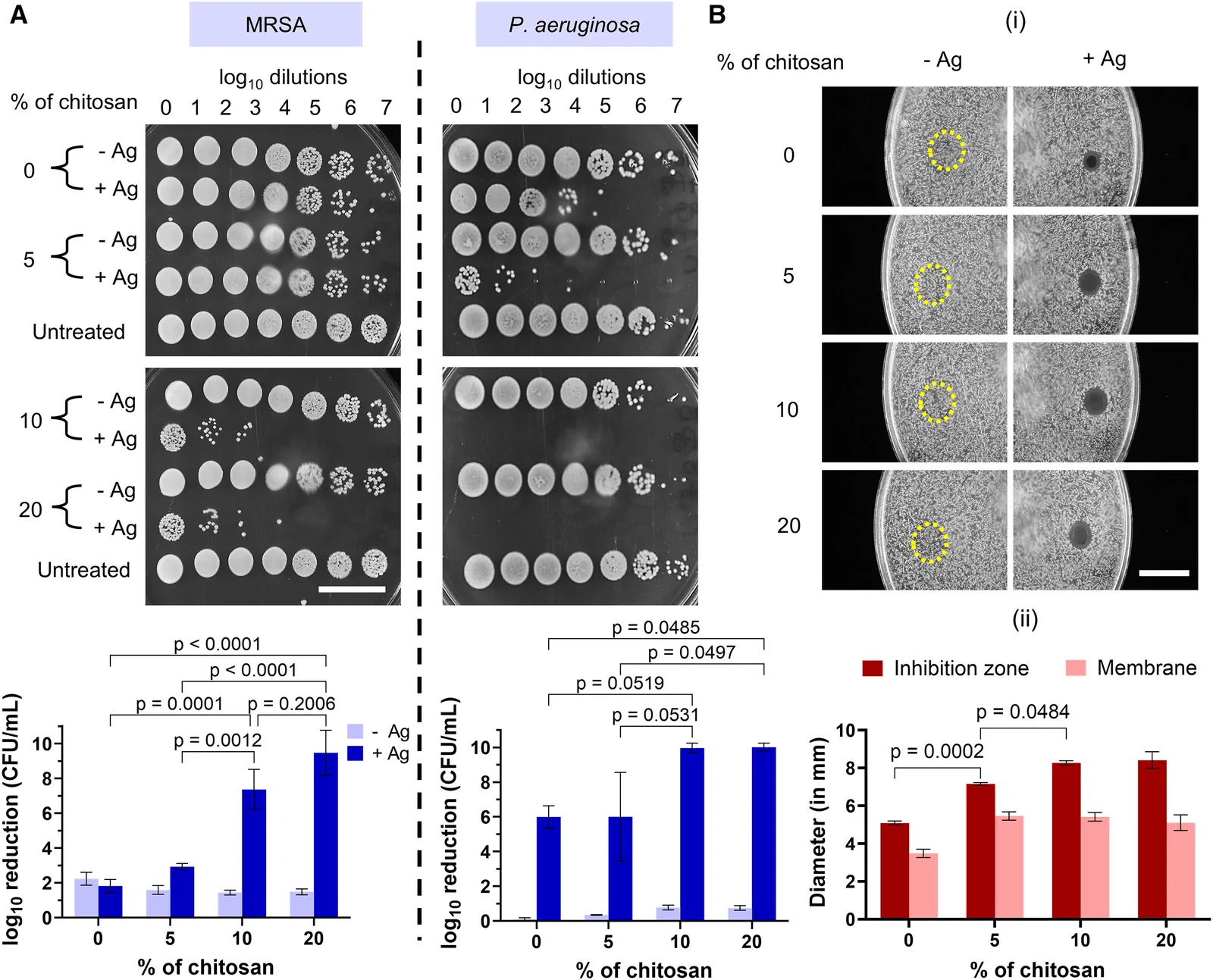
*In vitro* antibacterial efficacy of the electrospun membranes (A) *In vitro* evaluation of the antibacterial activity of the electrospun membranes after incubation with MRSA and *P. aeruginosa* for 18 h. Dot spots of 10-fold dilutions of the bacterial cultures are shown along with quantification of the reduction in CFU count. (B) Disk diffusion method showing the inhibition zone for MRSA after incubation with the membranes for 18 h. Data are represented as mean ± SEM (3 independent biological replicates, each with 3 technical replicates) (two-way repeated-measures ANOVA with Šidák’s multiple-comparisons test). Scale bar, 2 cm.

Several mechanisms have been proposed for the antibacterial activity of Ag^+^ ions which include (1) altering the permeability of the bacterial membrane, leading to the release of membrane proteins and intracellular molecules; (2) inducing oxidative damage to cellular structures; and (3) disrupting intracellular adenosine triphosphate (ATP) production and deoxyribonucleic acid (DNA) replication.^33^^,^^34^^,^^35^ In gram-positive bacteria such as MRSA, a cell wall composed of a thick peptidoglycan layer (approximately 30 nm) renders them less vulnerable to penetration by Ag^+^ ions than gram-negative bacteria such as *P. aeruginosa* having a thinner peptidoglycan layer (3–4 nm).^36^ In addition, gram-negative bacteria possess negatively charged lipopolysaccharides in the cell membrane, which promote the adhesion of Ag^+^ ions, thereby increasing their susceptibility to antibacterial activity. This explains the notably higher antibacterial effect of the membranes against *P. aeruginosa* than MRSA in Figure 4A. TEM and SEM images of MRSA after incubation with the membranes corroborate these observations, with the bacterial cells exhibiting morphology characteristic of damaged cells (see SI, Figures S9 and S10).

Interestingly, the growth curves of MRSA show that although the membranes had a 10-log reduction in antibacterial activity, the bacteria that remained grew more than the untreated ones in the presence of fresh media (see SI, Figure S11A). However, this population was still susceptible to the antibacterial activity of the membranes (see SI, Figure S11B).

The Kirby-Bauer disk diffusion method is a widely utilized technique for assessing bacterial susceptibility to antibiotics. We employed this method to evaluate the antibacterial activity of the membranes on solid culture media, which better simulates a clinical environment. Figure 4B shows the inhibition zone for MRSA after incubation with the membranes for 18 h as per the disk diffusion method. Here, a clear inhibition zone can be observed around the membranes, indicating the antibacterial effect of diffused Ag^+^ ions, attributed to their high diffusivity, rather than direct bacterial contact with the membranes or diffused Ag/SiO_2_ NPs.^37^ Although all membranes initially had a diameter of 6 mm, they shrank upon absorbing moisture, resulting in a reduced diameter.

Many studies utilize silver nitrate (AgNO_3_) as the source of Ag^+^ ions.^10^^,^^38^ Thus, we substituted Ag/SiO_2_ NPs with AgNO_3_ (maintaining the silver content) during electrospinning and studied the antimicrobial activity of these membranes (see SI, Figure S12). Membranes with AgNO_3_ exhibit slightly lower Ag^+^ release and comparable antibacterial activity to those with Ag/SiO_2_ NPs. CS in the polymer matrix reduces AgNO_3_ to silver NPs upon contact with water, raising toxicity concerns, favoring our original design with Ag/SiO_2_ NPs. In studies where the reduction to NPs is curtailed, a burst release of Ag^+^ has been reported, leading to toxicity.^11^ Compared with AgNO_3_-based membranes of matched silver content, the Ag/SiO_2_ system showed slightly higher Ag^+^ release with comparable antibacterial activity while avoiding the risk of *in situ* silver nanoparticle formation in the CS-containing matrix. This provides an important design advantage, as the flame-made, silica-stabilized Ag/SiO_2_ NPs can be incorporated in a controlled manner and remain retained within the fibers while still enabling sustained antibacterial Ag^+^ delivery.

We conducted further antibacterial studies using an *ex vivo* model developed on thawed porcine and fresh human skin, as illustrated in Figure 5A. Wounds were created on the skin and infected with bacteria at a concentration of 10^5^ CFUs per wound. The bacteria were allowed to colonize the wounds for 2 h, followed by a 4-h treatment with the membranes. Figure 5B displays dot spots of 10-fold dilutions of MRSA bacterial culture alongside the quantification of CFU reduction on thawed porcine skin. Membranes containing 10% and 20% CS were selected for this experiment, as they were the most effective configurations according to *in vitro* studies shown in Figure 4. The membranes demonstrated antibacterial activity even in the rigorous *ex vivo* model, which is highly conducive to bacterial growth, with the membrane containing 20% CS achieving a 3-log reduction (99.9%) in CFUs.

**Figure 5:**
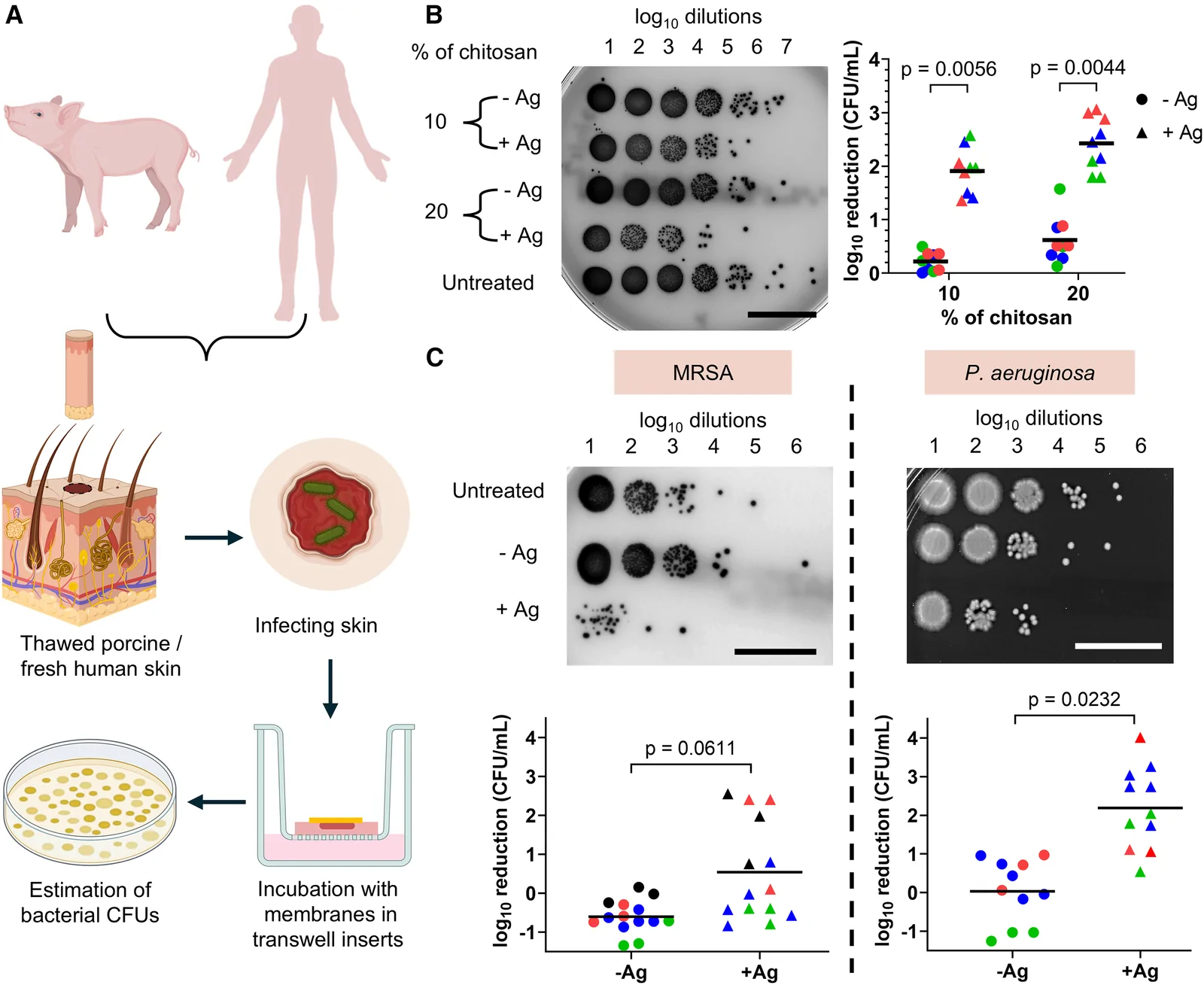
*Ex vivo* antibacterial efficacy of the electrospun membranes Schematics showing the workflow for *ex vivo* evaluation of antibacterial activity (A). The tests were performed on (B) thawed porcine skin infected with MRSA (*n* = 3) (two-way repeated-measures ANOVA with Šidák’s multiple-comparisons test) and (C) fresh human skin infected with MRSA (*n* = 4) and *P. aeruginosa* (*n* = 3) (two-tailed paired *t* test). Membranes containing 20% CS with and without Ag/SiO_2_ NPs were used on the human skin. Dot spots of 10-fold dilutions of the bacterial culture are shown along with quantification of the reduction in CFU count. Samples from the same donor are denoted by the same color. The solid black line refers to the mean value. (*n* is the number of independent donors, each with 3 biopsies). Scale bar, 2 cm.

The membrane containing 20% CS was subsequently selected for antibacterial studies on freshly excised human skin (from plastic surgery donors) with MRSA and *P. aeruginosa* as depicted in Figure 5C. Human skin varies in terms of age, gender, anatomical location, and biological factors, which significantly affect susceptibility to infections and antibacterial agents. This accounts for the considerable inter-donor and intra-donor variability shown in Figure 5C. Nevertheless, the membranes achieved a 1-log reduction (90%) in CFUs with MRSA and a 2-log reduction (99%) with *P. aeruginosa*. Testing wound dressings on *ex vivo* human skin is crucial as it is more physiologically relevant to real-life scenarios than porcine or rodent-based animal models.^39^ These experiments provide initial evidence of antibacterial activity in human skin tissue and support further preclinical evaluation of the developed membranes.

### Biocompatibility and wound healing

The toxic effects of wound dressings on human skin are a significant concern and a bottleneck in the production of any type of dressing.^16^^,^^40^ Failure to address this issue contributes to the high withdrawal frequency of dressings during clinical trials. Figure 6A outlines the experimental design for the biocompatibility study of electrospun membranes on freshly excised human skin. Wounds were created on punch biopsies of human skin and treated with the membranes for 18 h. After membrane removal, histological analysis was performed on cross-sections of the biopsies to observe wound characteristics. Figure 6B shows histological images of untreated samples and samples treated with membranes containing Ag/SiO_2_ NPs with 0% and 20% CS. The disruption of the epidermis at the center of the image indicates the wound.

**Figure 6:**
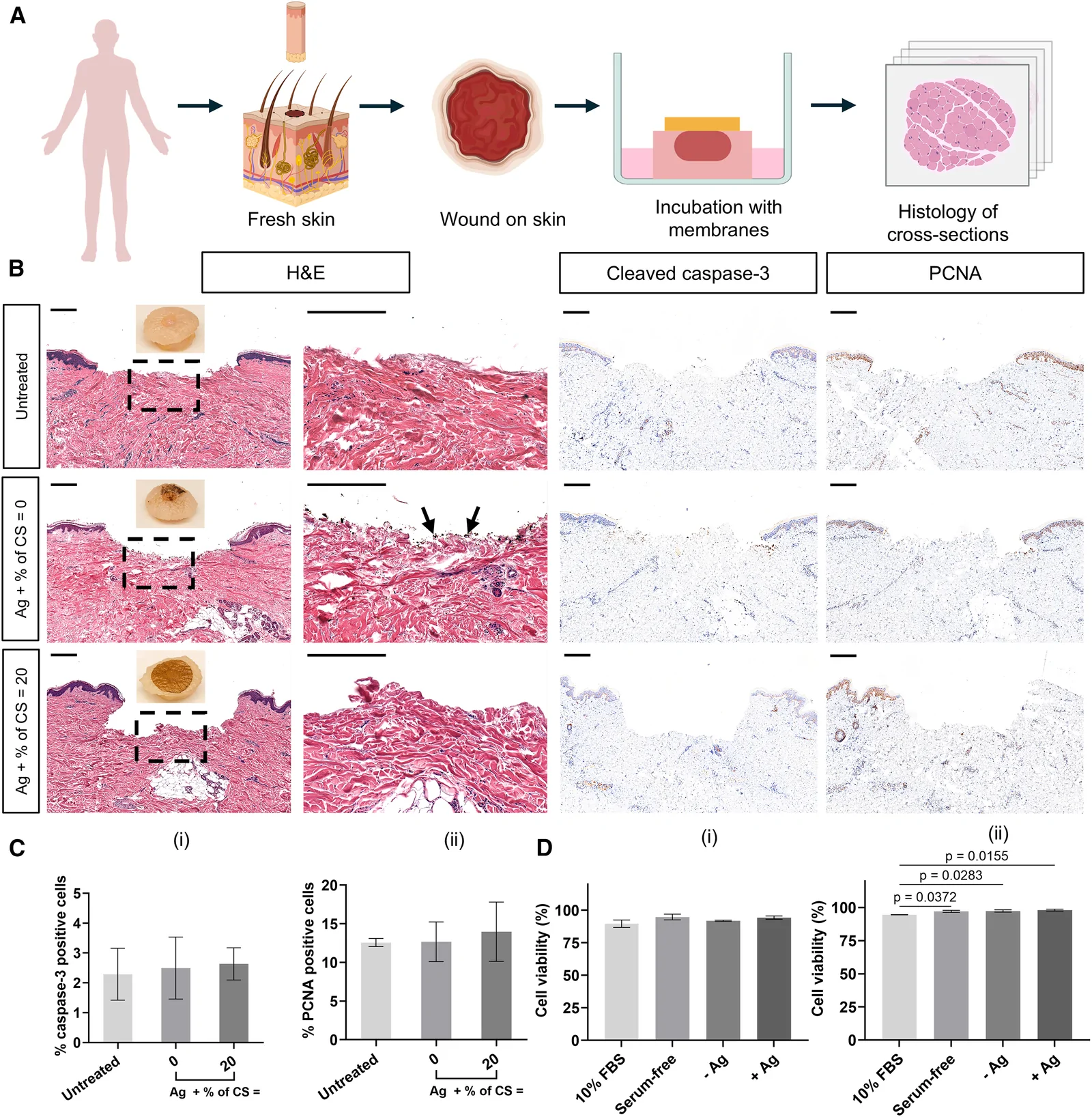
Biocompatibility and cytocompatibility studies on electrospun membranes (A) Schematic showing the workflow for *ex vivo* evaluation of biocompatibility on fresh human skin. (B) Images from H&E staining and immunohistochemistry of the same tissue 18 h after application of membranes containing Ag/SiO_2_ NPs with 0% and 20% CS. The images show the center of the wound with intact epidermis on either side. The outlined region in H&E-stained images is magnified where the black arrows denote Ag/SiO_2_ NPs on the wound surface. Scale bar, 300 μm. (C) Quantification of cells expressing (i) cleaved caspase-3 and (ii) PCNA from immunohistochemistry images. Data represented as mean ± SEM (1 donor, 3 biopsies). (D) Cell viability of (i) keratinocytes and (ii) fibroblasts, 72 h after incubation with extracts of membranes containing 20% CS with and without Ag/SiO_2_ NPs. The cell viability was evaluated using a lactate dehydrogenase assay. Data are represented as mean ± SEM (2 independent biological replicates, each with 3 technical replicates) (one-way repeated-measures ANOVA with Tukey’s multiple-comparisons test).

Hematoxylin and eosin (H&E) staining revealed no pathological changes in any sections, with the tissue showing normal architecture, including a well-differentiated epidermis composed of keratinized and stratified epithelium, and an underlying dermis with dense connective tissue. However, for the membrane containing Ag/SiO_2_ NPs with 0% CS, released NPs were visible on the wound (denoted by black arrows) as the membrane dissolved upon contact with the moist wound surface. This observation is also supported by Figure 3A, which shows the complete dissolution of this membrane in water, thereby releasing the particles. In contrast, no nanoparticle release was detected from membranes containing Ag/SiO_2_ NPs with 20% CS under the tested conditions, which may reduce nanoparticle accumulation and thereby mitigate associated long-term toxicity concerns.^18^

Immunohistochemistry was performed on these sections against cleaved caspase-3, indicative of cell apoptosis,^41^ and proliferating cell nuclear antigen (PCNA), denoting proliferating cells.^42^ The percentage of cells positive for these antigens is quantified in Figure 6C (see SI, Figure S13). A low number of cleaved caspase-3 positive cells was observed, while PCNA-positive cells were largely concentrated in the epithelium; both being characteristic of healthy tissue.

The electrospun membranes used in this study contain Ag/SiO_2_ NPs that release silver ions (Ag^+^), the active component responsible for antibacterial activity. Silver ions are known to cause toxicity to skin cells at higher concentrations.^11^^,^^12^ To evaluate this effect, extracts of the membranes were prepared in serum-free media according to the International Organization for Standardization (ISO) 10993-12 protocol.^43^^,^^44^ These extracts were tested against primary cells-human adult epidermal keratinocytes (HEKa) and human adult dermal fibroblasts (HDFa) using a lactate dehydrogenase (LDH) assay. LDH is a cytoplasmic enzyme released into the extracellular environment in the event of cell damage or lysis and serves as a marker for cytotoxicity.^45^^,^^46^ Figure 6D shows the viability of keratinocytes and fibroblasts after incubation with the membrane extracts for 72 h. All experimental groups exhibited approximately 90% cell viability, indicating no substantial cytotoxicity under the tested conditions.

We next investigated the influence of electrospun membranes on wound healing in human skin. Figure 7A illustrates the experimental design for this evaluation. Membranes containing 20% CS, with and without Ag/SiO_2_ NPs, were compared against untreated skin and a commercially available silver-based wound dressing (see SI, Figure S15 for details). Wounds were created on fresh skin biopsies, and the treatments were applied for 18 h. Figure 7Bi shows the wound surface after 4 days of incubation using Cell Tracker Green 5-chloromethylfluorescein diacetate (CMFDA) dye. The initial wound boundary is indicated by a dotted line, and the wound surface shows the current wound boundary and re-epithelialization. Tissue contraction, re-epithelialization, and wound closure are quantified from these images as shown in Figure 7Bii and iii. Wound closure in superficial wounds on human skin primarily involves re-epithelialization rather than tissue contraction,^47^ as evidenced by Figure 7Biii. The electrospun membranes exhibited comparable performance to untreated skin in the assessed wound-healing parameters. The delayed initiation of healing observed with the membranes is likely due to the mechanical impact of membrane removal on re-epithelialization. Nonetheless, the low tensile adhesion strength of these membranes on porcine skin suggests that they would not significantly interfere with the re-epithelialization process (see SI, Figure S14).

**Figure 7:**
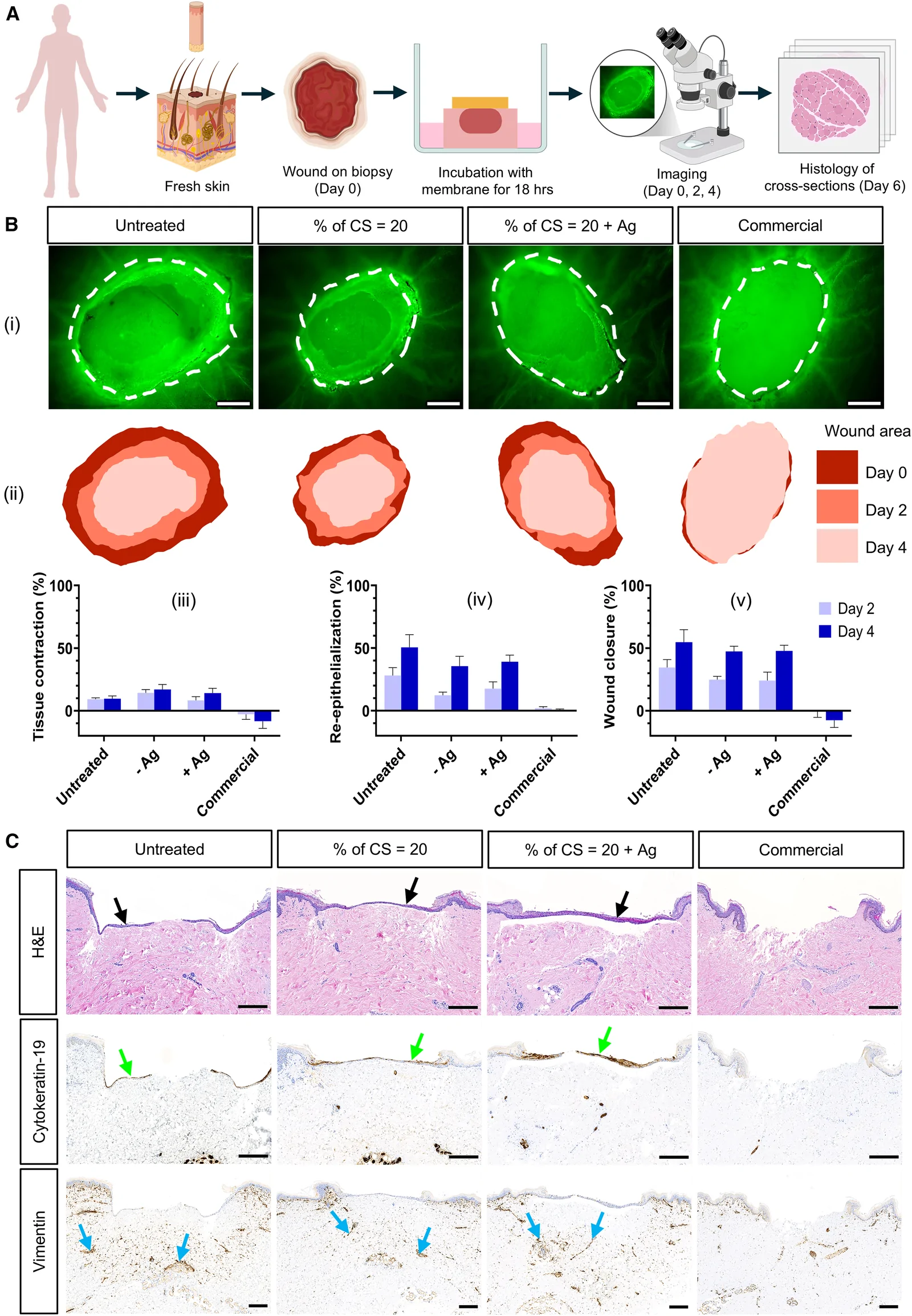
Influence of electrospun membranes vs. commercial dressing on wound healing (A) Schematic showing the workflow for *ex vivo* comparison of wound healing on fresh human skin in the presence of an electrospun membrane containing Ag/SiO_2_ NPs with 20% CS vs. a commercial silver-based wound dressing. (B) (i) Fluorescence microscopy images of the wound surface at day 4 using Cell Tracker^TM^ Green CMFDA dye. The dotted line represents the initial wound boundary. Scale bar, 500 μm. (ii) Representation of wound closure at day 0, day 2, and day 4. (iii) Tissue contraction, (iv) re-epithelialization, and (v) wound closure calculated from the microscopy images. Data are represented as mean ± SEM (1 donor, 4 biopsies). (C) Images from H&E staining and immunohistochemistry of the same tissue at day 6, showing distribution of cytokeratin-19 and vimentin. Black arrows denote re-epithelialization and wound closure. Green arrows denote upregulated cytokeratin-19 at the leading edge of the epithelium. Blue arrows denote fibroblasts expressing vimentin. Scale bar, 300 μm.

These observations are further validated by histological analysis of biopsy cross-sections, as shown in Figure 7C. H&E staining of these sections reveals the tissue architecture, while immunohistochemistry was conducted against cytokeratin-19, indicative of proliferating keratinocytes,^48^ and vimentin, denoting activated fibroblasts.^49^ Untreated skin and skin treated with the electrospun membranes exhibited characteristic features of healing tissue, including re-epithelialization and wound closure. Cytokeratin-19 is upregulated at the leading edge of the epithelium, indicating migrating keratinocytes, and at the outer root sheath of hair follicles. In addition, a significant number of fibroblasts in the dermis express vimentin, suggesting granulation tissue formation.

Under the tested *ex vivo* conditions, the commercial dressing was associated with reduced wound-healing responses compared with the untreated and electrospun-membrane groups, as evidenced by the lack of tissue contraction and re-epithelialization observed in Figure 7B, as well as the absence of proliferating keratinocytes and activated fibroblasts in Figure 7C. Additionally, the commercial dressing exhibited cytotoxicity toward fibroblasts despite having antibacterial activity only slightly higher than that of the electrospun membrane (see SI, Figure S15). The mechanistic focus of the present study is on the materials-level basis of performance, namely, nanoparticle retention, Ag^+^ release behavior, and healing compatibility, rather than on downstream molecular signaling pathways in skin cells. Detailed investigation of growth factor regulation, inflammatory signaling, and other molecular wound-healing mechanisms will be an important subject of future studies.

This study highlights the potential of membranes incorporating flame-made Ag/SiO_2_ NPs within electrospun PVA/CS fibers as effective antibacterial wound dressings. The fabrication processes, FSP and electrospinning, are well-established industrial techniques, highlighting the feasibility, scalability, and reproducibility of these membranes. Extensive characterization revealed the nanofibrous structure and uniform nanoparticle distribution within the fibers. The investigation into the silver ion (Ag^+^) release mechanism demonstrated a high and sustained release profile from a minimal amount of silver, without nanoparticle release. This contributes to significant antibacterial activity against gram-positive MRSA and gram-negative *P. aeruginosa*, which are key pathogens in wound infections. Additionally, the optimal Ag^+^ ion release profile was compatible with the cytocompatibility and short-term *ex vivo* human-skin responses observed under the tested conditions, supporting further evaluation of these membranes in preclinical models. Compared to other studies on electrospun membranes with Ag NPs (see SI, Table S1), this work comprehensively evaluates antibacterial efficacy through various microbiological assays. Although some studies report larger inhibition zones via disk diffusion, our system showed favorable cytocompatibility and no evident adverse tissue response in the short-term *ex vivo* human-skin experiments.

The observed performance of our membranes stems from the FSP-derived Ag/SiO_2_ NPs, which are sub-10 nm, with high surface area, and silica-stabilized to prevent aggregation. This enables uniform nanoparticle embedding during electrospinning, promoting sustained silver ion release without burst effects or mechanical defects. Crucially, strong nanoparticle-polymer interactions resulted in nanoparticle release not being detected, potentially overcoming a key limitation of many silver-based dressings.^11^^,^^12^

While FSP is an established technique for synthesizing NPs, embedding FSP-derived Ag NPs into electrospun wound dressings in a way that prevents their release opens up new directions in this field, potentially mitigating long-term toxicity concerns and differentiating this system from existing silver dressings. Furthermore, the dual role of CS as both a structural stabilizer (preventing PVA dissolution) and a biochemical modulator (enhancing swelling and reducing pH to increase Ag^+^ ion release) is demonstrated, enabling sustained antibacterial ion release from very low silver doses (∼20 μg/cm^2^), a key advance in biomaterials that aim to minimize cytotoxic thresholds while retaining therapeutic function. The novelty of the present work does not lie in claiming electrospinning, silver-based wound dressings, or a new antibacterial pathway as new per se, but in the integration of flame-made, silica-stabilized sub-10 nm Ag/SiO_2_ NPs into a PVA-CS electrospun matrix that enables sustained Ag^+^ release at low silver loading without detectable nanoparticle release from the CS-containing fibers. Together with the *ex vivo* validation of antibacterial efficacy and wound-healing compatibility in freshly excised human skin, this constitutes a meaningful materials-engineering and translational advance over conventional silver-loaded electrospun dressings. Finally, although *in vitro* antibacterial assays and *in vivo* murine models are routinely employed in the literature, *ex vivo* models using freshly excised human skin evaluating both antibacterial efficacy and wound healing histologically are rare and methodologically demanding. The present findings therefore extend the characterization of the developed dressings to human skin tissue and provide a basis for subsequent *in vivo* and preclinical evaluation.

### Limitations of the study

The present study was designed as a focused evaluation of antibacterial performance, nanoparticle retention, Ag^+^ release behavior, cytocompatibility, and short-term wound-healing compatibility in a clinically relevant *ex vivo* human skin model, rather than as a complete preclinical assessment. Accordingly, long-term toxicity, inflammatory profiling, collagen deposition, angiogenesis, extended Ag^+^ release, and comprehensive mechanical characterization remain important subjects for future *in vivo* studies.

## Resource availability

### Lead contact

Requests for further information and resources and data should be directed to and will be fulfilled by the lead contact, Georgios A. Sotiriou (georgios.sotiriou@su.se).

### Materials availability

This study did not generate new unique reagents.

### Data and code availability

• All the data reported in this paper will be shared by the lead contact upon request.
• This paper does not report original code.
• The protocol used for generating bivariate histograms from electron tomographs is detailed in the SI. Additional information is available from the lead contact upon request. Any additional information required to reanalyze the data reported in this paper is available from the lead contact upon request.

## Acknowledgments

This work received funding from the 10.13039/501100000781European Research Council (ERC) (no. 758705), the Karolinska Institutet Board of Research, the Swedish Foundation for Strategic Research (SSF) (FFL18-0043 and RMX18-0043), the 10.13039/501100004359Swedish Research Council (nos. 2021-05494 and 2023-03057), and the Strategic Research Area in Stem Cell Research and Regenerative Medicine (StratRegen) at 10.13039/501100004047Karolinska Institutet. The authors thank Birgitta Henriques-Normark, Staffan Normark, and the BHN group (KI) for the insightful discussions. The Karolinska Institutet 3D-EM, EMil, and BIC facilities are kindly acknowledged for the use of their equipment for TEM, SEM, and light microscopy image acquisition. FENO and Histocore facilities at Karolinska Institutet are appreciated for histological staining of the samples. The authors also thank Vitalii Shtender from Uppsala Universitet for conducting the ICP-OES analysis of the samples. The illustrations were created in Blender 2.8 and BioRender.

## Author contributions

R.V.R. was involved in conceptualization, synthesis and characterization, data curation, data collection, formal analysis, methodology, visualization, and writing and editing the original draft. J.G. was involved in cell culture for cytocompatibility studies, acquiring the images for wound healing, and sourcing the human skin for biocompatibility studies. M.S. was involved in developing the *ex vivo* wound infection model. T.T. was involved in EDX/EELS mapping and electron tomography as well as related visualization, methodology, analysis, and writing. M.F. was involved in XPS data acquisition as well as related visualization, and analysis. P.S. provided the human skin from plastic surgeries. N.X.L. was involved in the supervision of J.G. and result discussions. G.A.S. was involved in conceptualization, supervision, formal analysis, funding acquisition, methodology, resources, visualization, and writing and editing the original draft. All authors reviewed the results and approved the final version of the manuscript.

## Declaration of interests

G.A.S. is a co-founder of Aetherphlox AB working in the field of medical devices.

## STAR★Methods

### Key resources table

**Table:** 

REAGENT or RESOURCE	SOURCE	IDENTIFIER
**Antibodies**		

Cleaved caspase-3 antibody	Cell Signaling Technology	Cat# 9661; RRID: AB_2341188
PCNA antibody	Cell Signaling Technology	Cat# 13110; RRID: AB_2636979
Cytokeratin 19 antibody	Abcam	Cat# ab76539; RRID: AB_1523469
Vimentin antibody	Cell Signaling Technology	Cat#5741; RRID:AB_10695459

**Bacterial and virus strains**		

Methicillin-resistant *Staphylococcus aureus* MRSA USA300	ATCC	ATCC BAA-1680
*Pseudomonas aeruginosa* PAO1	ATCC	ATCC 15692

**Biological samples**		

Fresh human abdominal skin biopsies	Plastic surgery donors	Ethics approval# 2019–02335
Full-thickness porcine skin	Local breeder (stillborn piglets)	N/A

**Chemicals, peptides, and recombinant proteins**		

Silver acetate	Thermo Scientific Alfa Aesar	11311778; CAS: 563–63-3
Acetonitrile	Thermo Scientific Alfa Aesar	11394358; CAS: 75–05-8
2-Ethylhexanoic acid	Sigma-Aldrich	538701; CAS: 149–57-5
Hexamethyldisiloxane (HMDSO)	Sigma-Aldrich	205389; CAS: 107–46-0
Poly(vinyl alcohol), M_w_ 146-186k, D_H_ 88% (PVA)	Sigma-Aldrich	363103; CAS: 9002–89-5
Chitosan, medium M_W_	Sigma-Aldrich	448877; CAS: 9012–76-4
Tween 20	Sigma-Aldrich	P1379; CAS: 9005–64-5
Glycerol	Sigma-Aldrich	G5516; CAS: 56–81-5
Acetic acid, glacial	Sigma-Aldrich	695092; CAS: 64–19-7
Glutaraldehyde	Sigma-Aldrich	G5882; CAS: 111–30-8
Paraformaldehyde	Sigma-Aldrich	158127; CAS: 30525–89-4
Osmium tetroxide	Sigma-Aldrich	75632; CAS: 20816–12-0

**Critical commercial assays**		

LDH Cytotoxicity Detection Kit	Roche	11644793001
CoraLite Plus 488-Annexin V and PI Apoptosis Kit	Proteintech	PF00005

**Experimental models: Cell lines**		

Primary human epidermal keratinocytes (HEKa)	Thermo Fisher Scientific	C0055C
Primary human dermal fibroblasts (HDFa)	Thermo Fisher Scientific	C0135C

**Software and algorithms**		

ImageJ	NIH	https://imagej.nih.gov/ij/
GraphPad Prism	GraphPad	N/A
SerialEM	SerialEM	https://bio3d.colorado.edu/SerialEM/
IMOD	University of Colorado	https://bio3d.colorado.edu/imod/
MATLAB	MathWorks	N/A
Amira	Thermo Fisher Scientific	N/A
PDXL2	Rigaku	N/A
Kratos ESCApe	Kratos Analytical	N/A
QuPath 0.5.1	QuPath	https://qupath.github.io/

**Other**		

Fluidnatek LE-50 electrospinning system	Bioinicia	N/A
Talos 120C G2 TEM	Thermo Fisher Scientific	N/A
Themis Z TEM/STEM	Thermo Fisher Scientific	N/A
Aquilos 2 SEM	Thermo Fisher Scientific	N/A
Rigaku MiniFlex 600 XRD	Rigaku	N/A
Kratos AXIS Supra+ XPS	Kratos Analytical	N/A
Mettler Toledo perfectION Ag/S electrode	Mettler Toledo	N/A
FastPrep-24 5G Homogenizer	MP Biomedicals	N/A

### Experimental model and study participant details

The gram-positive Methicillin-Resistant *Staphylococcus Aureus* (MRSA-ST8: USA 300 strain), and the gram-negative *Pseudomonas aeruginosa* (PAO1 strain) were cultured in Luria-Bertani (LB) broth. This process involved an overnight incubation at 37°C with a shaking speed of 220 rpm.

Full-thickness skin was collected from piglets of a local breeder, that were confirmed to be stillborn before collection. The animals were not bred, euthanized, or otherwise used for research purposes, and their death was unrelated to this study. On this basis, the use of the resulting postmortem skin was not considered an animal experiment requiring ethical approval under the applicable Swedish regulations, as confirmed by Swedish Board of Agriculture. The skin was stored at −20°C. Skin samples of 10 mm diameter were prepared from the thawed and sterilized porcine skin.The research protocol for *ex vivo* studies on human skin was approved by the Stockholm Regional Ethics Committee (2019–02335) and was executed in compliance with the guidelines of the Declaration of Helsinki. Experiments were performed on fresh human skin samples procured from abdominal plastic surgeries. All participants gave their written informed consent for the collection and use of their samples, and publication. Wounds were generated using a 2 mm biopsy punch on the epidermal region and excised with a 6 mm biopsy punch. These skin samples are referred to as ‘biopsies’ in this work.

### Method details

#### Fabrication of nanoparticles and nanofibers

##### Flame spray pyrolysis

Ag/SiO_2_ nanoparticles were synthesized using flame spray pyrolysis.^15^ Silver acetate (99%, Alfa Aesar) was dissolved at 90°C under reflux in a mixture of acetonitrile (≥99.5%, Alfa Aesar) and 2-ethylhexanoic acid (99%, Sigma Aldrich) in equal parts to form a 0.4 M silver solution. To this solution, hexamethyldisiloxane (HMDSO, ≥98%, Sigma Aldrich) was added to obtain a target weight content of 50% silica in the Ag/SiO_2_ nanoparticles. The solution was then introduced into a capillary (Kel F 7750–12, HAMILTON) at a rate of 3 mL/min using a syringe pump (New Era Pump Systems, Inc.). Oxygen (˃99.5%, Linde) was used to atomize the solution into fine droplets at a gas flow rate of 8 L/min and a pressure drop of 1.8 bar. These droplets were then ignited by a pilot flame, created by mixing methane (˃99.5%, Linde) and oxygen (˃99.5%, Linde) at flow rates of 1.5 L/min and 3.2 L/min, respectively. The resulting dry Ag/SiO_2_ nanoparticle powders were collected from the aerosol by vacuum filtration using water-cooled glass fiber filters (Hahnemühle, 257 mm, GF6) and a MINK vacuum pump (Busch).

##### Electrospinning

The nanoparticles were incorporated into nanofibers through electrospinning using a Fluidnatek LE-50 system (Bioinicia). A 7% w/v mixture of polyvinyl alcohol (PVA, D_H_ = 88%, Sigma Aldrich) and chitosan (CS, medium MW, Sigma Aldrich) was prepared in 2% v/v of acetic acid in water where the amount of CS was varied as 0, 5, 10, and 20% of the total amount of polymer. 1% v/v of Tween 20 (Sigma Aldrich) and 30% w/w of glycerol (Sigma Aldrich) were added to the mixture to act as surfactant and plasticizer, respectively. The mixture was heated at 90°C for 4 h and kept at ambient temperature overnight to facilitate dissolution and mixing. The solution was fed to the spinneret using a glass syringe (Poulten Graf) at 0.4 mL/h. The distance between the 16 Gauge needle and the spinning collector was fixed at 10 cm. The high voltage applied between the spinneret and the collector was adjusted to obtain a stable Taylor cone. This value varied from 10 kV for 0% CS to 18 kV for 20% CS. The spinneret was moved back-and-forth for a distance of 5 cm at 5 cm/s to have a larger area of deposition on the collector which was rotated at 200 rpm. The system was maintained at ambient temperature and 15% humidity.

#### Characterization of nanoparticles and nanofibers

##### Transmission electron microscopy

A nanoparticle dispersion was created at a concentration of 0.5 mg/mL in ethanol, using sonication for even distribution. Carbon-coated copper grids (FCF400, Electron Microscopy Sciences) were then dipped into the nanoparticle dispersion and allowed to dry at ambient temperature. The samples were subsequently imaged with a Talos 120C G2 microscope, equipped with a Ceta-D detector and a 120 kV LaB6 electron source. The acquired images were processed and analyzed with ImageJ software.

Nanofibers containing 20% CS were directly spun onto the carbon-coated copper grids for a duration of 10 s. Tomographic images were recorded by choosing a fiber aligned roughly parallel to the tilt axis of the TEM and acquiring BF-STEM images while tilting from −45° to +45°, with a tilt increment of 1°. Acquisition was performed using SerialEM.^50^ The alignment of the tilt series and 3D tomogram reconstruction was performed with the IMOD package,^51^ particle size analysis was performed using custom-written code in the MATLAB programming language, and 3D visualization was performed using the Amira software package (Thermo Fisher Scientific). Other nanofibers were directly spun onto copper grids (200 mesh, Agar Scientific) with a larger mesh size to facilitate tomography which, however, led to charging and distortion of images. To mitigate this, the samples were coated with a 10 nm layer of carbon using thermal evaporation of a carbon filament in a Leica EM ACE600 system. The remainder of the tomography workflow was unchanged as detailed in SI.

##### Scanning transmission electron microscopy

Scanning transmission electron microscopy (STEM) images and elemental mapping images of the aforementioned samples were captured using an aberration-corrected transmission electron microscope (Thermo Fisher Scientific^TM^ Themis Z, operating at 300 kV) equipped with a Super-X energy-dispersive X-ray (EDX) detector (Thermo Fisher) and a Quantum post-column Gatan Image Filter (Gatan Inc.). Elemental maps were produced using the hypermodal data fusion method, which amalgamates co-registered low-loss electron energy loss spectroscopy (EELS), core-loss EELS, and EDX datasets through weighted matrix concatenation which is detailed in the SI.

##### Scanning electron microscopy

The topographical features of the electrospun fibers were examined using a scanning electron microscope (Aquilos 2, Thermo Fisher Scientific) equipped with an Everhart-Thornley secondary electron (SE) detector. The observations were conducted at a magnification of 20000x, utilizing an operating voltage of 10 kV, a current of 13 pA, and a working distance of 7 mm. Before the imaging process, the samples were secured onto SEM stubs using carbon adhesive tape and were subjected to an *in-situ* sputter coating of platinum for a duration of 15 s, under an operating current of 30 mA and a pressure of 0.1 mbar.

##### X-ray diffraction

A compact desktop powder X-ray diffractometer (Rigaku MiniFlex 600) equipped with a Cu-K-α source was used to conduct X-ray diffraction (XRD) measurements. The 2-theta angle ranged from 10° to 80° with a scan rate of 1°/min and a step width of 0.01°. Diffraction data analysis was performed using the PDXL2 software to identify the elemental peaks.

##### X-ray photoelectron spectroscopy

X-ray photoelectron spectroscopy (XPS) analysis was conducted utilizing Kratos AXIS Supra+ X-ray photoelectron spectrometer furnished with an Al Kα source. The survey spectrum exhibited peaks attributable to silica, a recognized surface contaminant in XPS studies. To mitigate this issue, the samples were subjected to an etching process through sputtering with 10 keV Ar1000+ clusters for a duration of 20 s and a raster size of 2 × 2mm, prior to the spectroscopy. The spectrum for silver was analyzed and the Gaussian fits performed using Kratos ESCApe software. The area under the curves were then used to calculate the oxide to metal ratio.

##### Adhesion studies

The adhesion of the membranes to porcine skin was assessed using a texture analyzer TA XT.Plus (Stable Micro Systems). Porcine skin biopsies, 10 mm in diameter, were incubated at 37°C for 2 h in CO2 Independent Medium (Gibco) supplemented with 4 mM GlutaMAX (Gibco) and 10% FBS (Gibco). The biopsies were then mounted on a 15 mm diameter cylindrical stage. A membrane, 12 mm in diameter, was affixed to a 15 mm diameter cylindrical probe and applied to the porcine skin surface with a force of 0.1 N for 60 s. The system was configured to auto-trigger with a trigger force of 0.01 N. The pre-test, test, and post-test speeds were set to 1 mm/s, 0.5 mm/s, and 1 mm/s, respectively.

#### *In vitro* bioactivity analysis

##### Estimation of silver ion release

For these experiments, the fibers were cut into circular disks with a diameter of 12 mm. The release of Ag^+^ ions was quantitatively assessed using a Mettler Toledo electrode system (perferctION comb Ag/S). The fibers were weighed and then submerged in 3 mL of deionized water at ambient temperature. The electrode was calibrated at concentrations of 0.01, 0.1, and 1 mg/L, and measurements were taken at time intervals of 1, 5, 10, and 24 h. The profile of Ag^+^ ion release was normalized in accordance with the weight of the samples, which exhibited minor variations.

##### Inductively coupled plasma-optical emission spectroscopy

To determine the total quantity of silver, the fibers were weighed and dissolved in concentrated nitric acid (HNO_3_). The samples were subsequently diluted with 5% HNO_3_ and analyzed using Inductively Coupled Plasma-Optical Emission Spectroscopy (ICP-OES, Avio 200, PerkinElmer).

##### UV-vis spectrophotometry

Inorder to verify whether Ag/SiO_2_ NPs are released, the fibers were immersed in 3 mL of deionized water at ambient temperature for a duration of 24 h. Subsequently, 1 mL of the supernatant was utilized to acquire UV-visible absorbance spectra using a Specord 210 Plus UV-vis spectrophotometer system (Analytik Jena), spanning a wavelength range of 300–800 nm.

##### Swelling studies

To quantify the degree of swelling, the dry weight of the samples was initially determined. Subsequently, the samples were immersed in 1 mL of de-ionized water. After removal from the water, the samples were blotted with a filter paper to remove excess water, and the wet weight was then measured. This analysis was conducted solely on the samples containing 20% and 10% chitosan, as the remaining samples exhibited high brittleness and disintegrated upon immersion in water. The swelling percentage was subsequently calculated based on these measurements as

$$Swelling\;ratio\;\left(\%\right)=\frac{Wet\;weight-Dry\;weight}{Dry\;weight}\times 100$$

##### Estimation of pH

The pH values were determined using a Mettler Toledo pH meter (FiveEasy Cond meter F30). The fibers were immersed in 3 mL of de-ionized water for 24 h after which their pH was measured. The system was calibrated using standards of pH values 4.01,7 and 9.21 prior to use. The reduction in pH was calculated relative to de-ionized water.

#### *In vitro* antibacterial studies

##### Estimation of CFU count

The bacterial cultures were adjusted to 10^5^ CFUs/mL in LB. Membranes created by electrospinning, measuring 5 mm in diameter, were then sterilized under UV light for 30 min. For each experimental group, a pair of these membranes were placed in a 96-well plate in sets of three. Subsequently, 150 μL of the bacterial cultures were introduced to the wells and incubated at 37°C for 18 h. Following this, a 10-fold dilution sequence was prepared, and 10 μL of these dilutions were dotted onto Luria agar plates. These plates were then left to incubate overnight, after which the growth of bacterial colonies was both quantified and imaged.

##### Electron microscopy

Following an 18-h incubation with the membranes, the bacterial culture was centrifuged at 14,000 g for 2 min and resuspended in a fixation solution containing 2.5% glutaraldehyde and 1% paraformaldehyde in 0.1 M PBS for 2 h. The cells were subsequently washed with 0.1 M PBS and imaged using a Zeiss Ultra 55 scanning electron microscope. For transmission electron microscopy, the cells were post-fixed with 2% OsO_4_ in 0.1 M PBS for 2 h at 4°C, followed by a 2-h wash in PBS at 4°C. The cells were then dehydrated in 70% ethanol and 95% ethanol at 4°C for 30 min each, followed by absolute ethanol for 20 min and acetone for 30 min at room temperature. The samples were embedded in LX-112 resin by gradually increasing the resin-to-acetone ratio: 1:2 for 3 h, 1:1 overnight, 2:1 over day, and pure resin overnight. The resin was cured by heating at 60°C, sectioned, mounted on TEM grids, and imaged using a transmission electron microscope (FEI Tecnai G2 BioTWIN).

##### Resistance of MRSA toward silver

To investigate whether MRSA develops resistance toward activity of the membranes, the bacteria were initially incubated with the electrospun membranes for 18 h. The cultures were centrifuged at 14000g for 2 min to remove traces of the treatment. The bacteria were then resuspended in fresh LB with an initial OD_600_ of 0.003 and their growth was monitored for 18 h using OD_600_ measurements with a Bioscreen C instrument.

##### Disk diffusion studies

For Kirby-Bauer disk diffusion studies, 100 μL of 10^6^ CFUs/mL of MRSA in LB was plated on Mueller-Hinton agar plates. Sterilized membranes, measuring 6 mm in diameter, were then placed on the plates and incubated at 37°C for 18 h. The diameters of the inhibition zone and that of the membranes were measured using vernier calipers.

#### *Ex vivo* antibacterial studies on porcine skin

Dermal wounds were created using a rotary drill (Dremel) and infected with a 5 μL culture containing 10^5^ CFUs of MRSA in LB. The samples were then placed in transwell inserts (0.4 μm pore size, Millipore) and transferred to 12-well plates. Each well contained 1 mL of CO_2_ Independent Medium (Gibco) supplemented with 4 mM GlutaMAX (Gibco) and 10% FBS (Gibco). After 2 h of incubation at 37°C, sterilized electrospun membranes, measuring 12 mm in diameter, were placed on the skin. The larger diameter was to account for the immediate shrinkage of the membranes after coming in contact with the moist wound surface. The biopsies were treated with the membranes at 37°C for 4 h and then transferred to 2 mL tubes containing lysing matrix M (MP Biomedicals) and 500 μL PBS (Sigma Aldrich). Afterward, they were homogenized in a FastPrep-24 5G homogenizer (MP Biomedicals) at 6 m/s for 6 cycles of 20 s each. A 10-fold dilution sequence was then prepared from the lysate, and 10 μL of these dilutions were dotted onto MRSA-selective agar plates. These plates were incubated overnight, after which the growth of bacterial colonies was quantified and imaged.

#### *Ex vivo* studies on human skin

##### Sourcing human skin

Biocompatibility studies: Sterilized electrospun membranes, 8 mm in diameter, were applied to the wounds. The biopsies were incubated in 12-well plates containing media (Dulbecco’s Modified Eagle Medium (DMEM), supplemented with 10% fetal bovine serum (FBS), and antibiotics (penicillin 50 U/L and streptomycin 50 mg/mL (ThermoFisher Scientific)), at a temperature of 37°C, 5% CO_2_ for 18 h. Subsequently, the biopsies were fixed in 4% paraformaldehyde at 4°C for 24 h. After a brief wash with PBS, the biopsies were transferred to 70% ethanol, dehydrated in xylene, embedded in paraffin, sectioned using a microtome, and stained with hematoxylin and eosin. Immunohistochemistry was performed using an automated BOND RX^m^ (Leica Biosystems), with sections stained for cleaved caspase-3 (1:1000 μL), and proliferating cell nuclear antigen (PCNA, 1:2000 μL) (Cell Signaling Technology) and counterstained with DAB (3,3′-Diaminobenzidine). The slides were then imaged by a PANNORAMIC™ Digital Slide Scanner (3DHistech) and DAB-positive nuclei quantified using QuPath 0.5.1.

Wound healing studies: To evaluate wound healing, the wounds were treated with sterilized electrospun membranes, 8 mm in diameter, for 18 h in 8-well chamber slides (Ibidi Gmbh) containing media (Dulbecco’s Modified Eagle Medium (DMEM), supplemented with 10% fetal bovine serum (FBS), and antibiotics (penicillin 50 U/L and streptomycin 50 mg/mL (ThermoFisher Scientific)), at a temperature of 37°C, 5% CO_2_. The membranes were then removed and the biopsies incubated in the chamber slides with media at 37°C, 5% CO_2_ for one week. The wound surface was imaged using a stereo microscope (Leica Biosystems) at day 0 (prior to membrane application), and on days 2, 4, and 6. Cell Tracker^TM^ Green CMFDA (ThermoFisher Scientific) was used to visualize wound contraction and re-epithelialization, with the dye washed off using phosphate buffered saline (PBS) after use. The images were then analyzed using ImageJ to estimate the percentage of tissue contraction, re-epithelization, and wound closure. Histological analysis was performed on a representative biopsy from each group on day 6, as described above. Sections were stained with hematoxylin and eosin, and immunohistochemistry was performed using antibodies against cytokeratin 19 (1:1000 μL) (Abcam) and vimentin (1:1000 μL) (Cell Signaling Technology). The slides were then imaged by a ZEISS Axioscan 7 scanner.

Antibacterial studies: The biopsies were incubated overnight in 12-well plates containing media (CO_2_ Independent Medium (Gibco) supplemented with 4 mM GlutaMAX (Gibco) and 10% FBS (Gibco)), at a temperature of 37°C for acclimatization. The wounds were then infected with a 5 μL culture containing 10^5^ CFUs of MRSA or *P.aeruginosa* in LB. The biopsies were placed in transwell inserts (0.4 μm pore size, Millipore) and transferred to 12-well plates containing media. After 2 h of incubation at 37°C, sterilized electrospun membranes, measuring 8 mm in diameter, were placed on the skin. Rest of the protocol remained same as that for porcine skin.

Cytotoxicity studies: The reagents were obtained from ThermoFisher Scientific unless otherwise specified. Primary human adult epidermal keratinocytes (HEKa, Cat. No.- C0055C) were cultured in EpiLife medium, supplemented with 10% fetal bovine serum (FBS), 1% human keratinocyte growth supplement (HKGS) and 1% penicillin. Primary human adult dermal fibroblasts (HDFa, Cat. No. - C0135C) were cultured in Dulbecco’s Modified Eagle Medium (DMEM, ThermoFisher Scientific), supplemented with 10% fetal bovine serum (FBS), and antibiotics (penicillin 50 U/L and streptomycin 50 mg/mL). The cells were then seeded on 96-well microtiter plates at a density of 1000 and 2500 cells/well for HEKa and HDFa respectively. The cells were allowed to adhere and spread on the plate surface for 24 h at 37°C, 5% CO_2_. The media was then replaced with membrane extracts (100 μL/well) prepared by incubating 6 mm punch biopsies of each membrane in 500 μL of serum-free media (CO2 Independent Medium supplemented with 4 mM GlutaMAX) at 37°C for 18 h. Media with 10% FBS, which supports optimal cell proliferation, served as positive control while serum-free media was used to assess basal cell growth. The cells were incubated with the extracts for 72 h at 37°C after which the lactate dehydrogenase (LDH) release from the cells was determined using a cytotoxicity detection kit (Roche). A positive control was included in the LDH assay using 1% Triton X-100 to achieve complete cell lysis and determine the maximum releasable LDH activity. This value was used as a reference to calculate percentage cytotoxicity, as per manufacturer’s instructions.

For microscopy, HDFa cells were seeded on 8-well chamber slides at a density of 7500 cells/well. After 24 h of adhesion and spreading at 37°C in a 5% CO_2_ atmosphere, the medium was replaced with membrane extracts (300 μL/well) and incubated for 72 h at 37°C. The cells were then labeled for apoptotic and dead cells using the CoraLite Plus 488-Annexin V and PI Apoptosis Kit (Proteintech) according to the manufacturer’s instructions.

### Quantification and statistical analysis

All statistical analyses were performed using GraphPad Prism software. Depending on the experimental design, statistical significance was assessed using two-way repeated-measures ANOVA followed by Šidák’s multiple-comparisons test, one-way repeated-measures ANOVA followed by Tukey’s multiple-comparisons test, or a two-tailed paired *t* test. Quantitative data are presented as mean ± standard error of the mean (SEM), except where otherwise indicated; Ag^+^ ion release data are presented as mean ± standard deviation (SD). Exact *p* values are reported in the corresponding figures. The statistical test used, exact value of n, definition of n (including the number of independent biological replicates, donors, biopsies, membrane samples, and/or technical replicates, as applicable), and measures of center and dispersion are provided in the corresponding figure legends.
