# Supplementary material for: Chitosan-stabilized electrospun nanofiber dressings with embedded flame-made nanosilver for the treatment of resistant infections
Source: iScience. 2026 Oct 23;29(11):117991. doi: 10.1016/j.isci.2026.117991 (PMC13720824; doi:10.1016/j.isci.2026.117991)
Supplement: Supplementary file 1 [file mmc1.pdf]

iScience, Volume *29*

Supplemental information

Chitosan-stabilized electrospun nanofiber dressings with embedded flame-made nanosilver for the treatment of resistant infections

Reshma V. Ramachandran, Jennifer Geara Semaan, Maria Samara, Thomas Thersleff, Matthew Fielden, Pehr Sommar, Ning Xu Landén, and Georgios A. Sotiriou

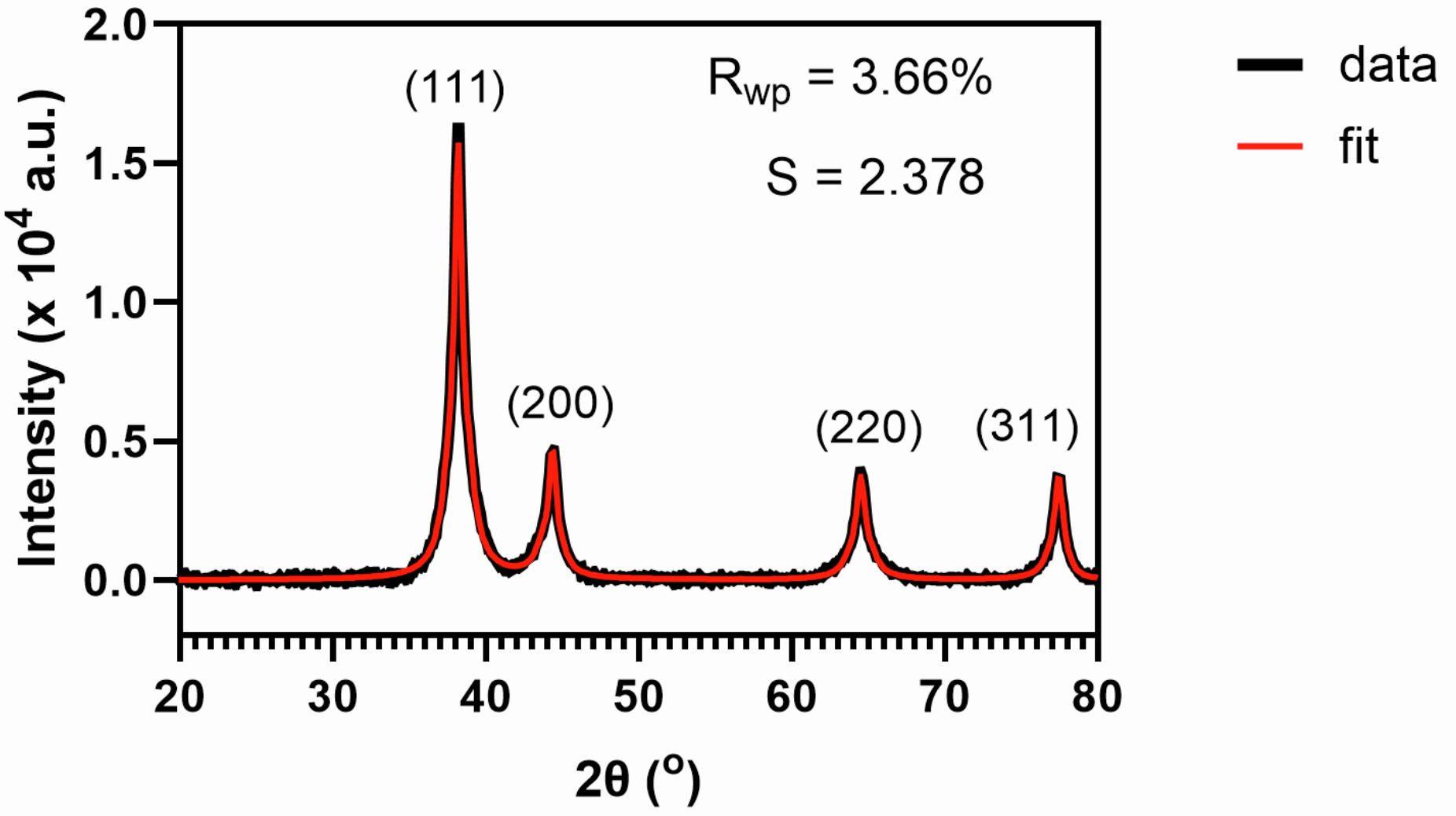

Figure S1: Diffractogram of Ag/SiO_2 NPs with the peaks corresponding to silver annotated. The red line shows the fitting model used to calculate crystal size as per Rietveld analysis. R_{wp} denotes the weighted difference between observed and calculated intensities and the low value indicates a good fit. S denotes the goodness of fit and S > 1 would denote that the model does not fully capture the sample characteristics.

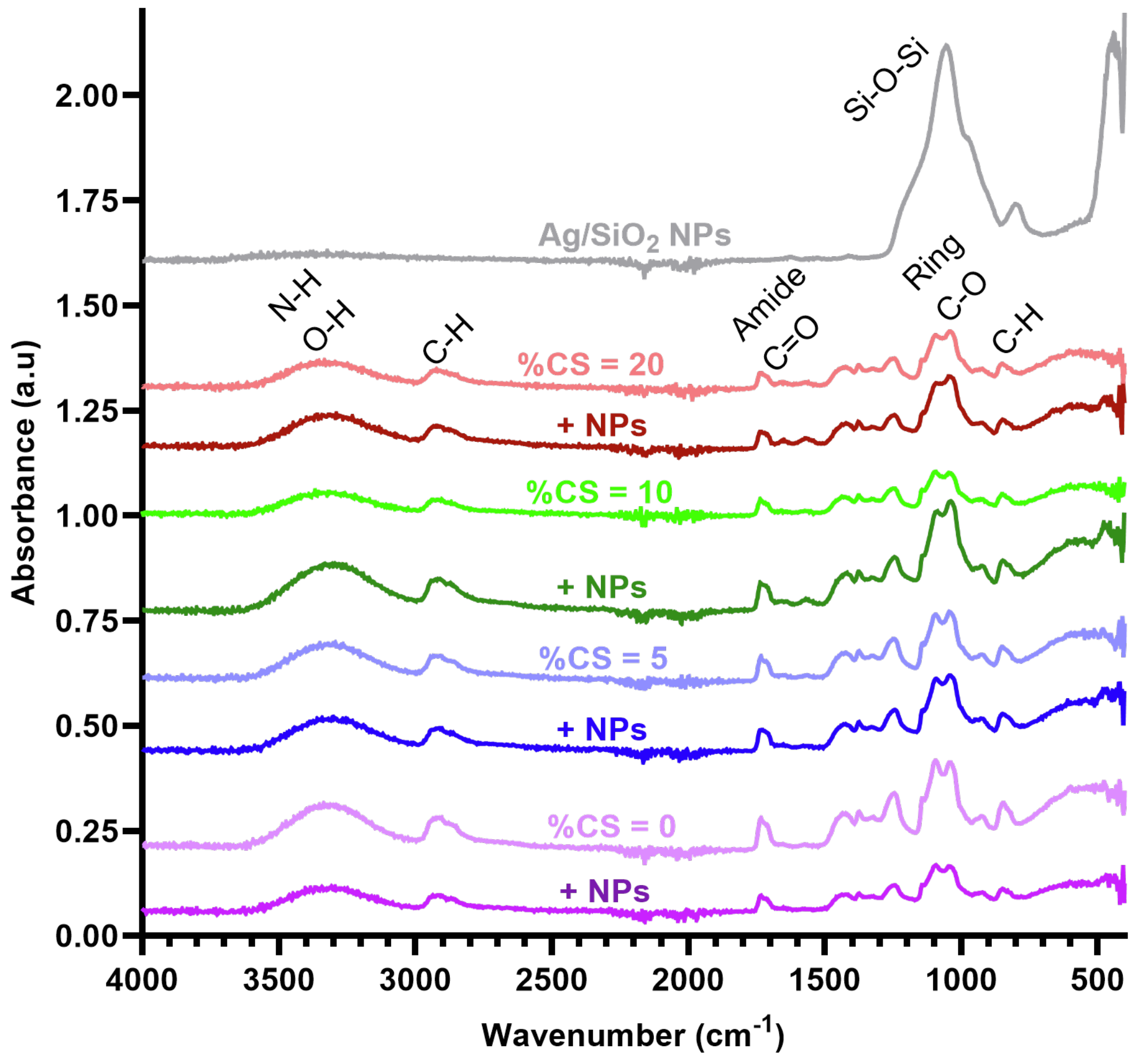

Figure S2: FTIR spectra of the electrospun membranes containing various amounts of chitosan with and without Ag/SiO_2 NPs. The characteristic peaks of polyvinyl alcohol (PVA) and chitosan (CS) are annotated.

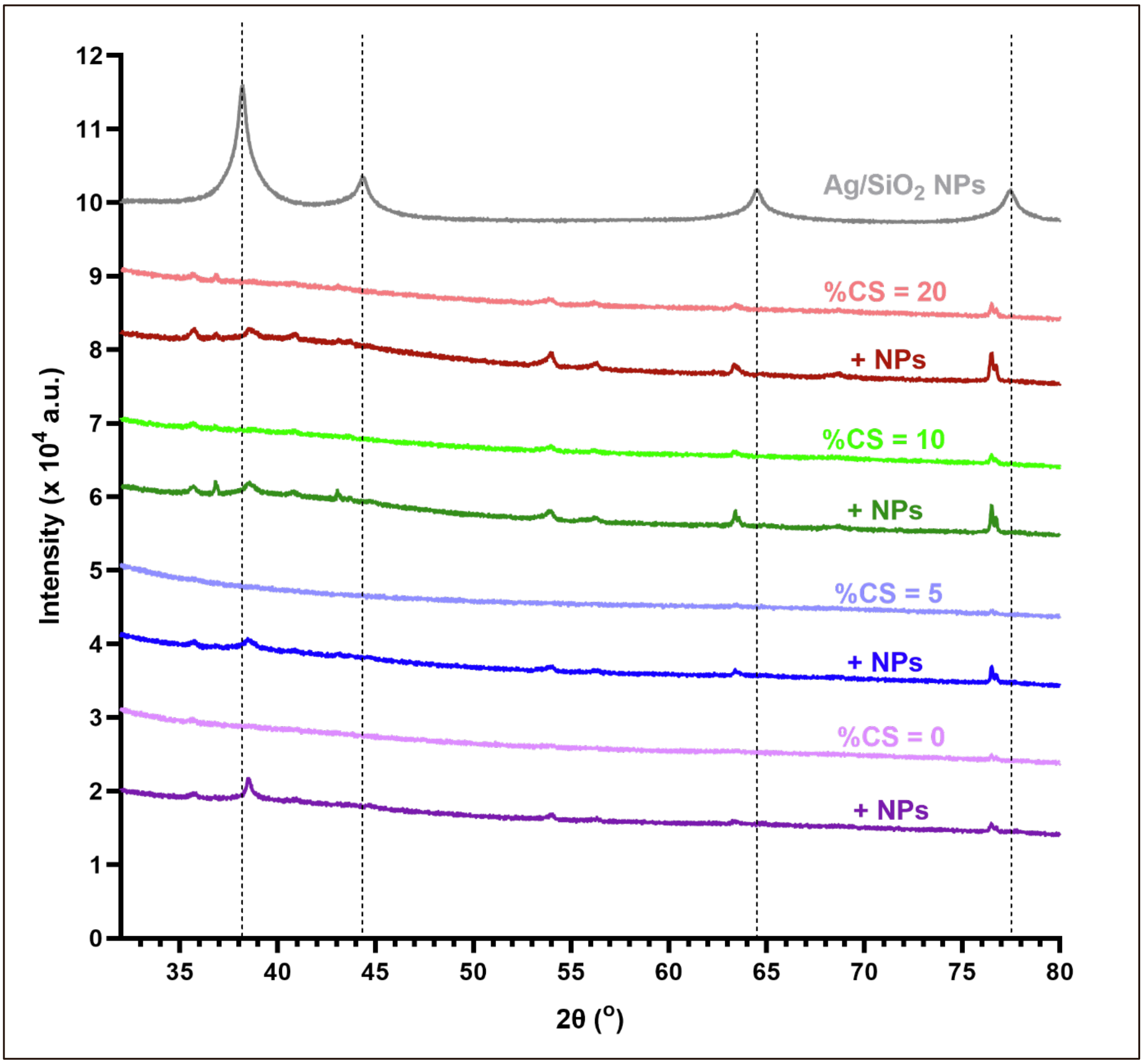

Figure S3: Diffractograms of electrospun membranes containing various amounts of chitosan (CS) with and without Ag/SiO_2 NPs. The characteristic silver peak at 38.18° is seen. The slight increase in the angle may be attributed to uneven adhesion of the membranes to the sample holder.

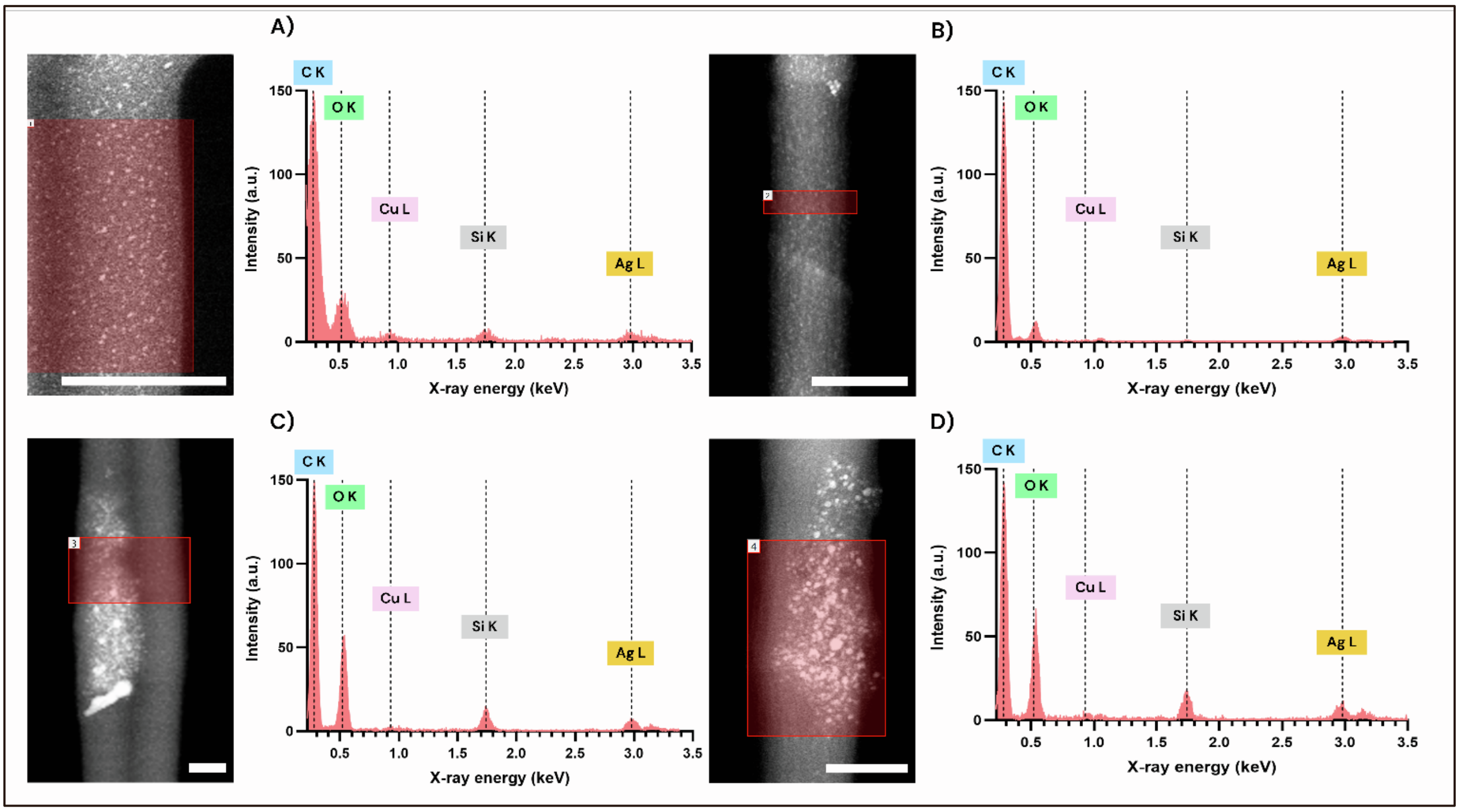

Figure S4: HAADF-STEM images of electrospun membranes containing Ag/SiO_2 NPs. Amount of chitosan varies as (A) 20% (B) 10% (C) 5% and (D) 0%. The EDX spectrum of the outlined region is also shown for all the samples with annotations for various elements. Hydrocarbons account for the C peak and some amount of the O peak. The rest of the O peak as well as the Si and Ag peaks can be attributed to the Ag/SiO_2 NPs in the membrane. The Cu peak can be traced to the copper grid used for supporting the fibers.

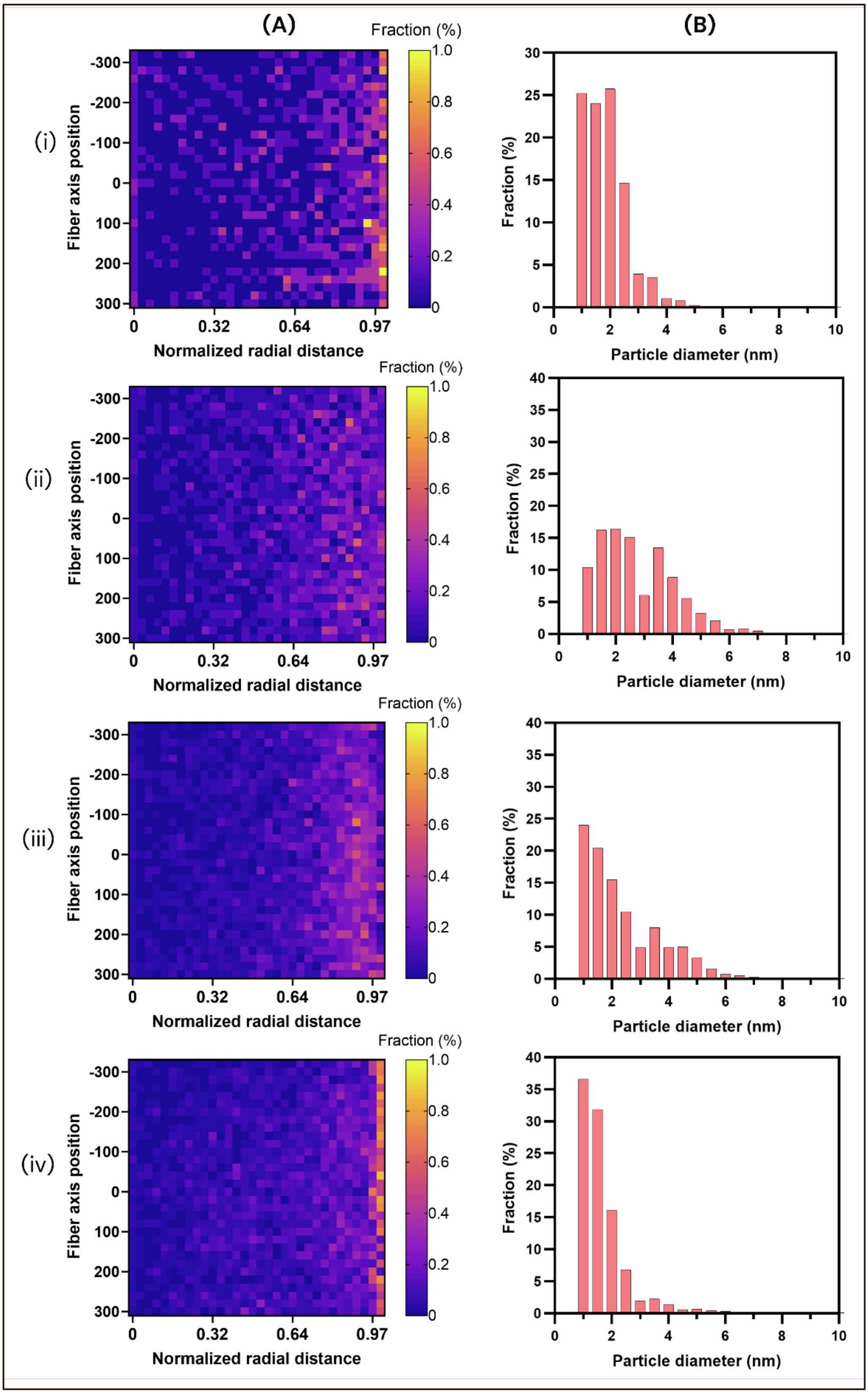

Figure S5: Bivariate histogram showing the nanoparticle frequency as a function of the normalized radial distance along the fiber axis position. Each histogram is derived from a single fiber of electrospun membrane containing Ag/SiO_2 NPs. The amount of chitosan in these membranes varies as (i) 20% (ii)10% (iii) 5% and (iv) 0%. (A) Radial distribution of NPs from different positions on the fiber axis. (B) Distribution of particle diameter.

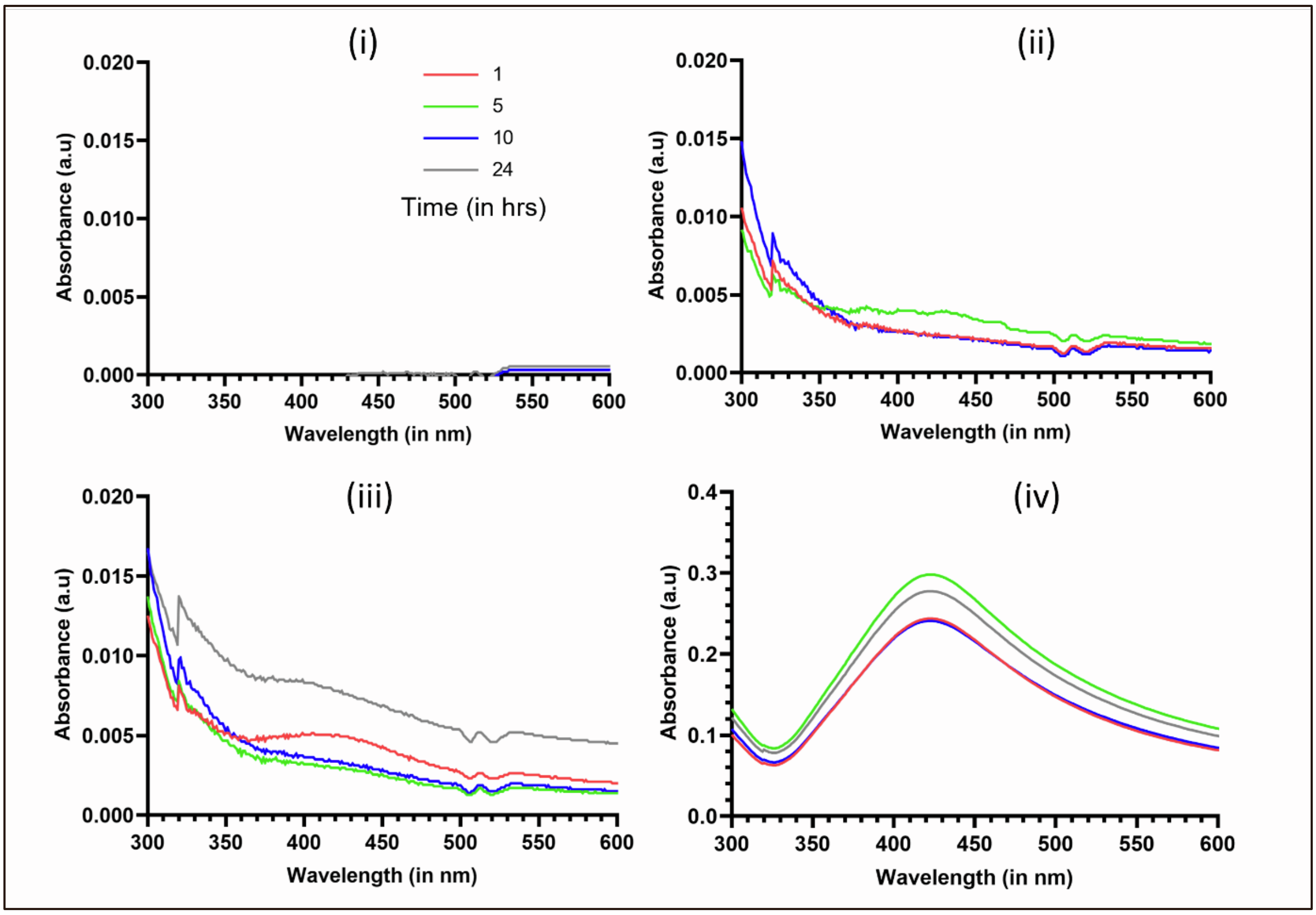

Figure S6: Absorbance of the electrospun membranes in the UV-visible range after immersion in de-ionized water for various intervals of time. The percentage of chitosan in these membranes are (i) 20, (ii) 10, (iii) 5, and (iv) 0. In the membrane with 0% CS, the peak at 420 nm in the membrane corresponds to the surface plasmon resonance of silver nanoparticles and is an indication of the release of Ag/SiO_2 NPs from the membrane

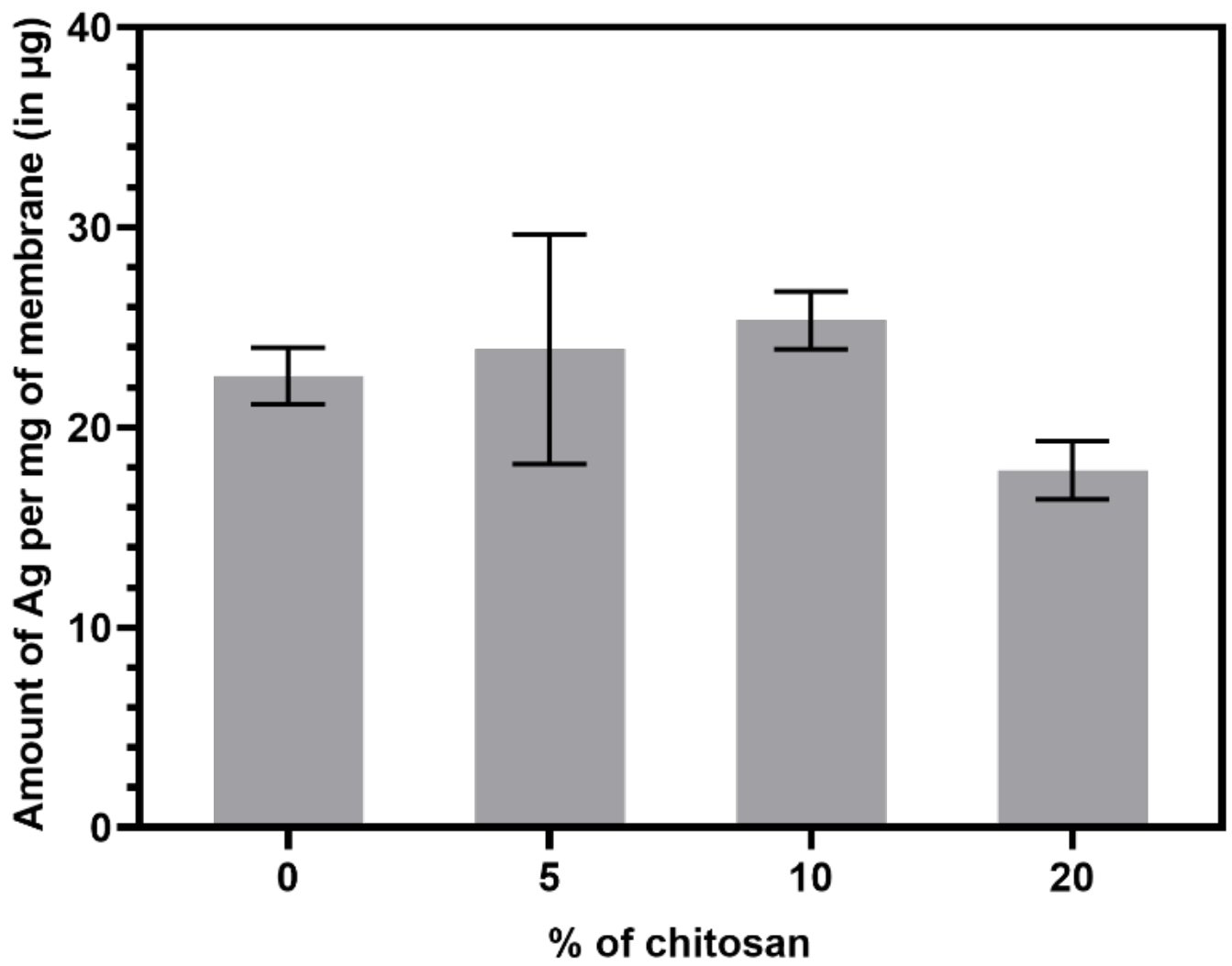

Figure S7: Amount of silver in 1 mg of electrospun membrane as detected by Inductively coupled plasma- atomic emission spectroscopy (ICP-OES). Data represented as mean ± SEM. (n=2).

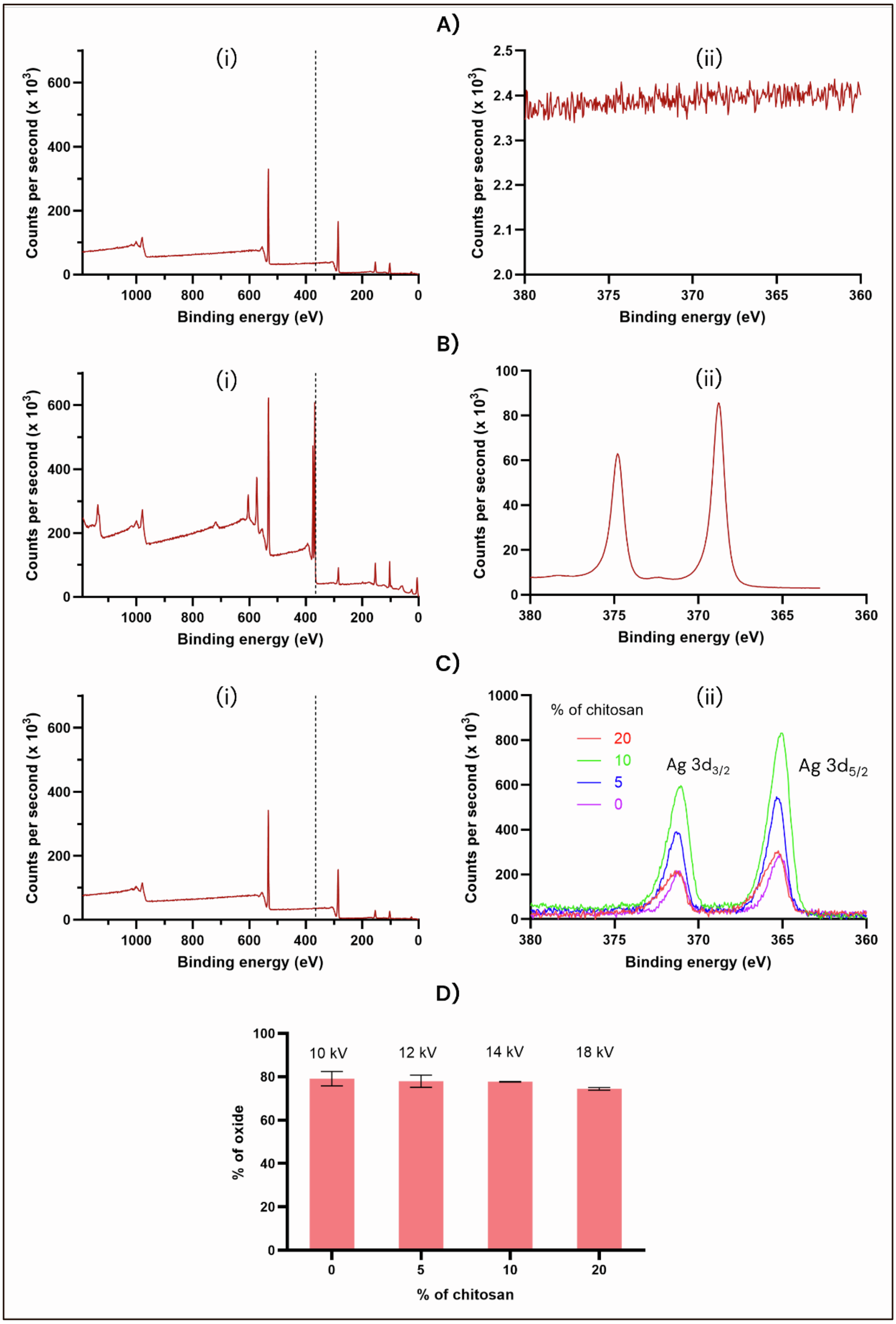

Figure S8: Estimation of silver oxide content in electrospun membranes containing Ag/SiO_2 NPs using X-ray photoelectron spectroscopy (XPS). Since the NPs are encapsulated in the fibers, the samples were etched to obtain the signal of silver. (A) Membrane containing 20% of chitosan without Ag/SiO_2 NPs. (B) Plain Ag/SiO_2 NPs. Survey spectra of unetched samples is shown in (i) and Ag 3d peaks of etched samples in (ii). (C)-(i) Survey spectrum of unetched membrane containing Ag/SiO_2 NPs and 20% of chitosan. (C)-(ii) Ag 3d peaks of etched membranes containing Ag/SiO_2 NPs and various amounts of chitosan. D) Amount of silver oxide present in the membranes as estimated from deconvolution of Ag $3d_{5/2}$ peak. Data represented as mean ± SD (N=3). The voltage (kV) used for electrospinning is also shown. With the deconvolution being limited by the low signal-to-noise ratio, significant difference was not observed between the amount of silver oxide in the samples.

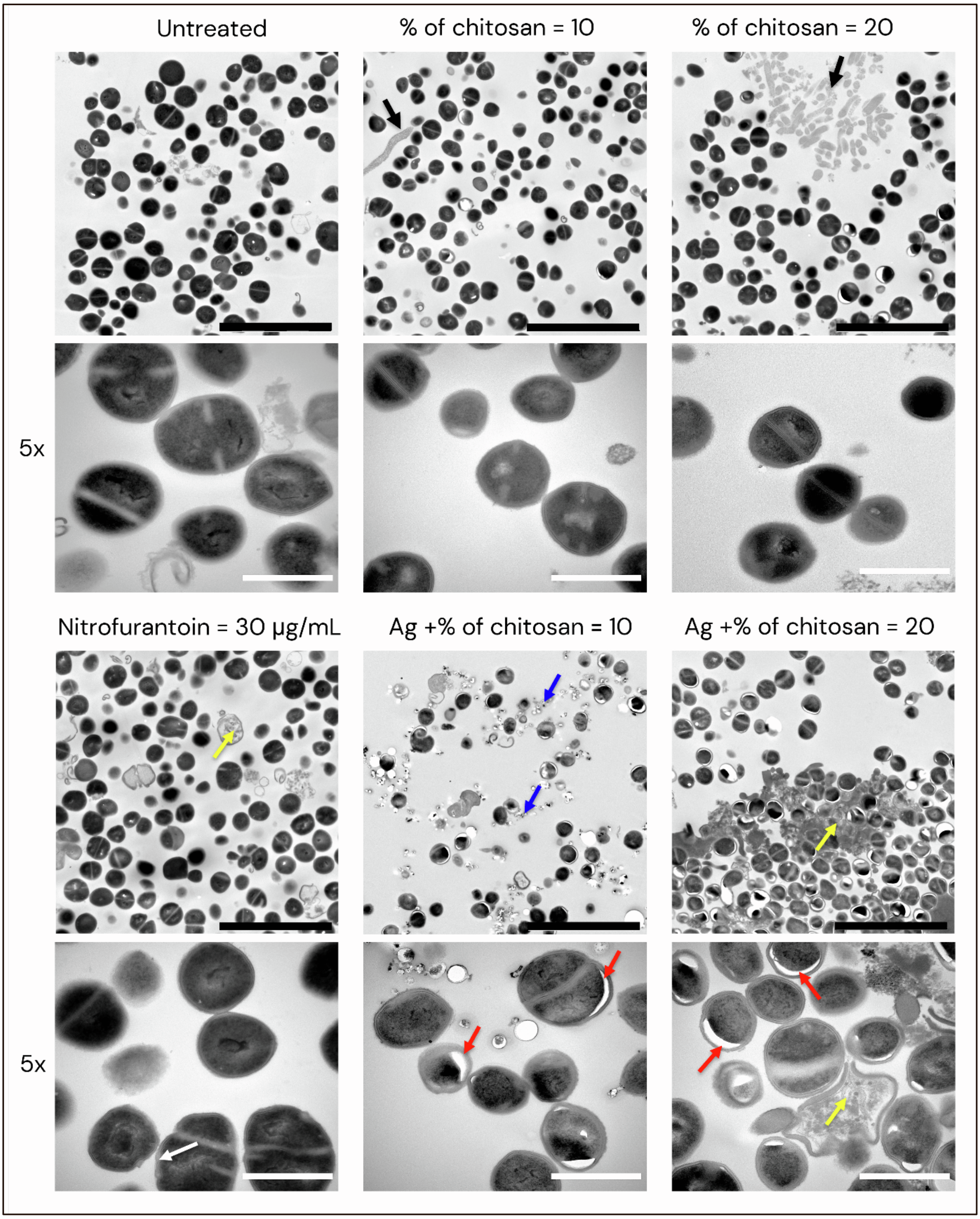

Figure S9: TEM images of MRSA post 18-hour incubation with electrospun membranes. Untreated cells and those incubated with membranes lacking Ag/SiO_2 NPs exhibited smooth spherical surfaces, indicative of healthy cells. Black arrows highlight fiber residues from the membranes, while yellow arrows indicate dead cells. Nitrofurantoin served as a positive control for cell death, given its mechanism of action is akin to that of Ag^+ ions. Here, the white arrow points to an undulating cell surface, suggesting a loss of outer membrane integrity. Blue arrows denote debris-like structures near the cells, and red arrows indicate the separation of the outer membrane from the cytoplasmic membrane, which may be due to cell death or artifacts from cell processing. The low number of bacteria in samples incubated with electrospun membranes containing Ag/SiO_2 NPs makes them particularly prone to processing artifacts. Nonetheless, these images support the in vitro results which demonstrate high antibacterial activity for silver-containing membranes. Scale bar = 5 μm and 1 μm (for 5x images)

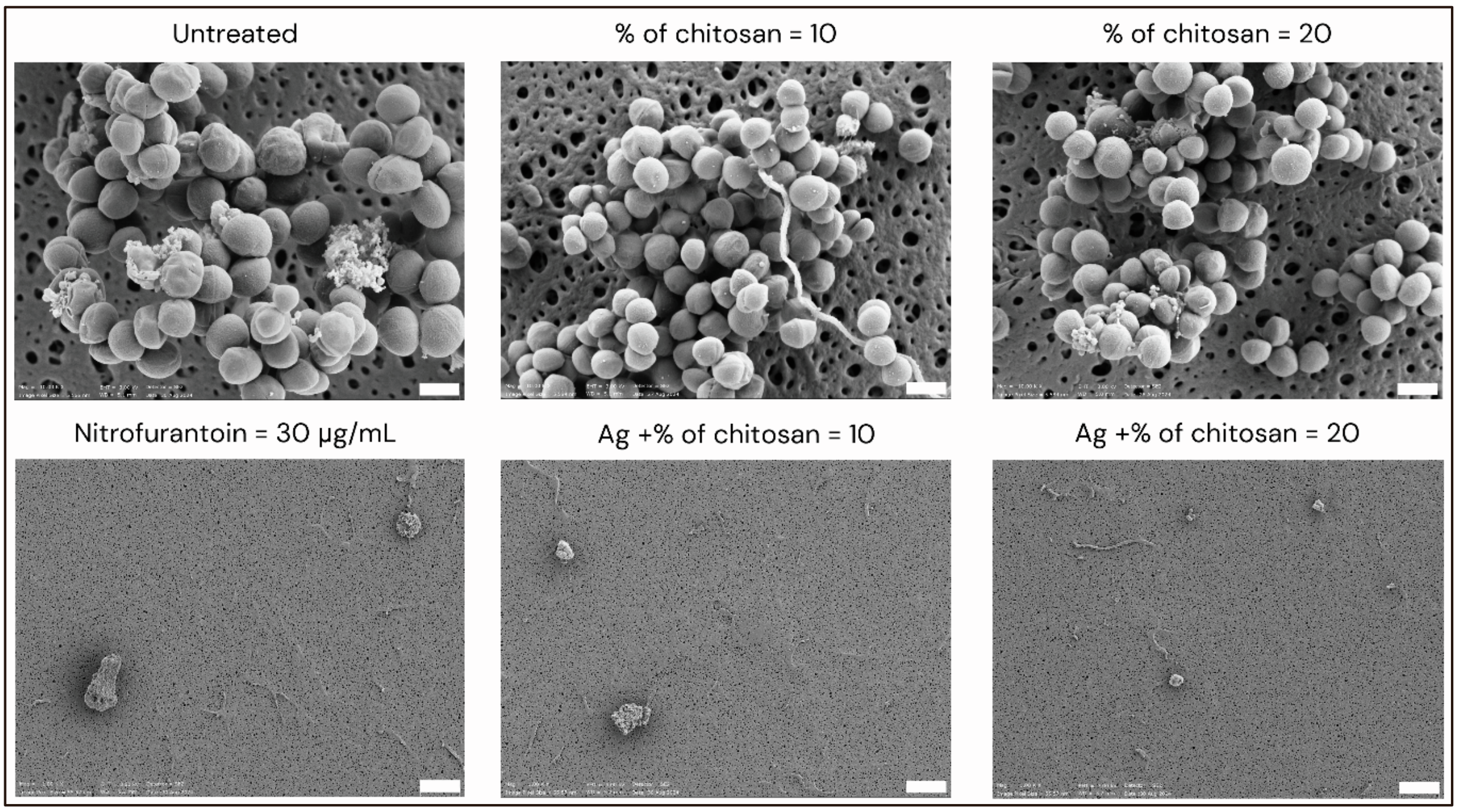

Figure S10: SEM images of MRSA post 18-hour incubation with electrospun membranes. Untreated cells and those incubated with membranes devoid of Ag/SiO_2 NPs display smooth, spherical surfaces with a uniform clustered arrangement, indicative of healthy cells. In contrast, samples treated with Nitrofurantoin (positive control for cell death) and electrospun membranes containing Ag/SiO_2 NPs exhibit no live cells, only cell debris. Scale bar= 1 µm (top row), 10 µm (bottom row).

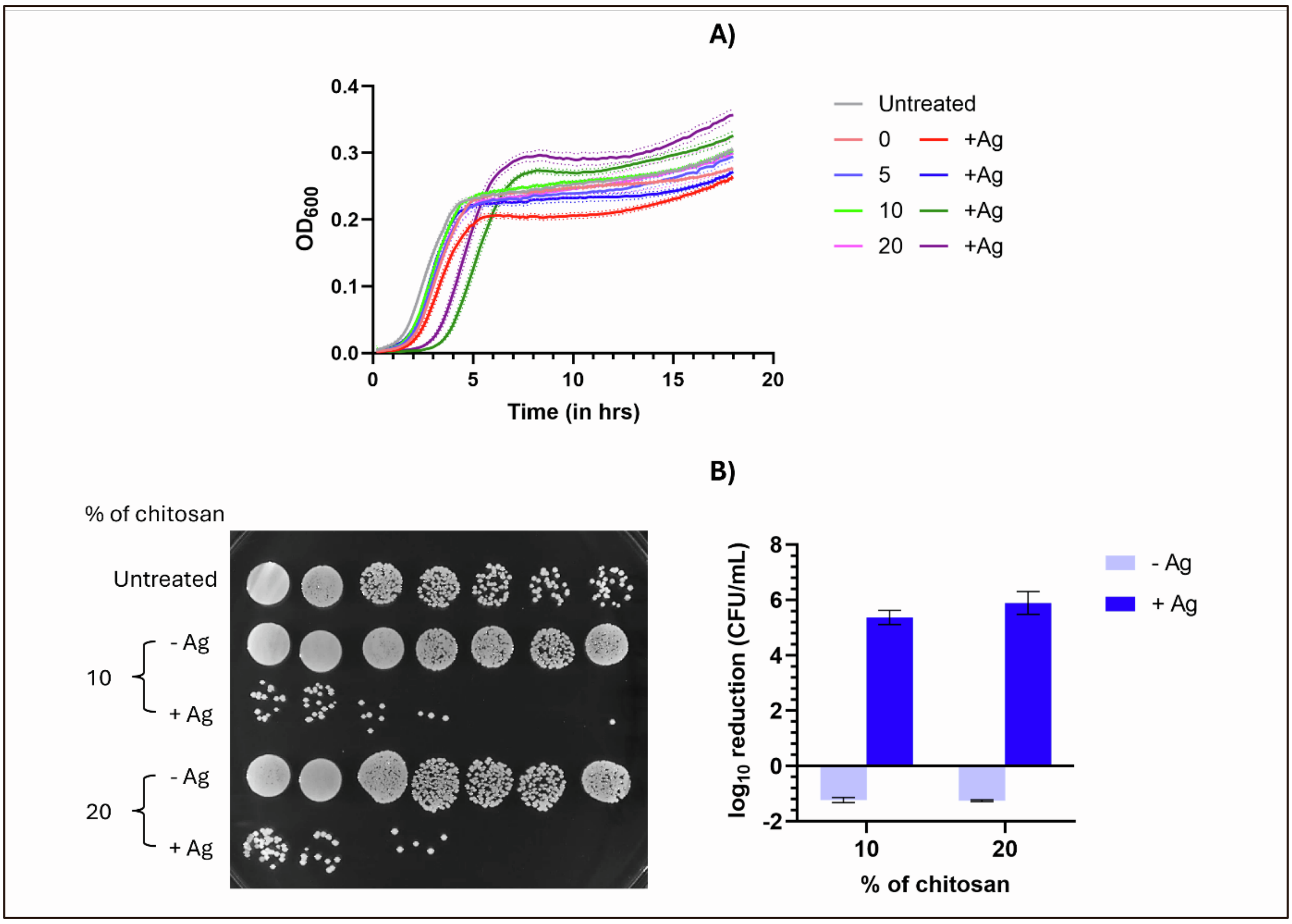

Figure S11: Antibacterial activity of electrospun membranes with pre-treated MRSA. A) Growth curves of MRSA that were pre-treated with membranes containing Ag/SiO_2 NPs and 0, 5, 10 or 20% of chitosan for 18 hrs. The cultures were then centrifuged to remove the treatment, and the bacterial growth was monitored in fresh media for 18 hrs. Dotted lines denote the error bars (SEM). B) In-vitro evaluation of antibacterial efficacy of the electrospun membranes after 18 hours of incubation with pre-treated MRSA. Dot spots of ten-fold dilutions of the bacterial culture are shown alongside quantification of the reduction in CFU count. Data represented as mean ± SEM (n=3)

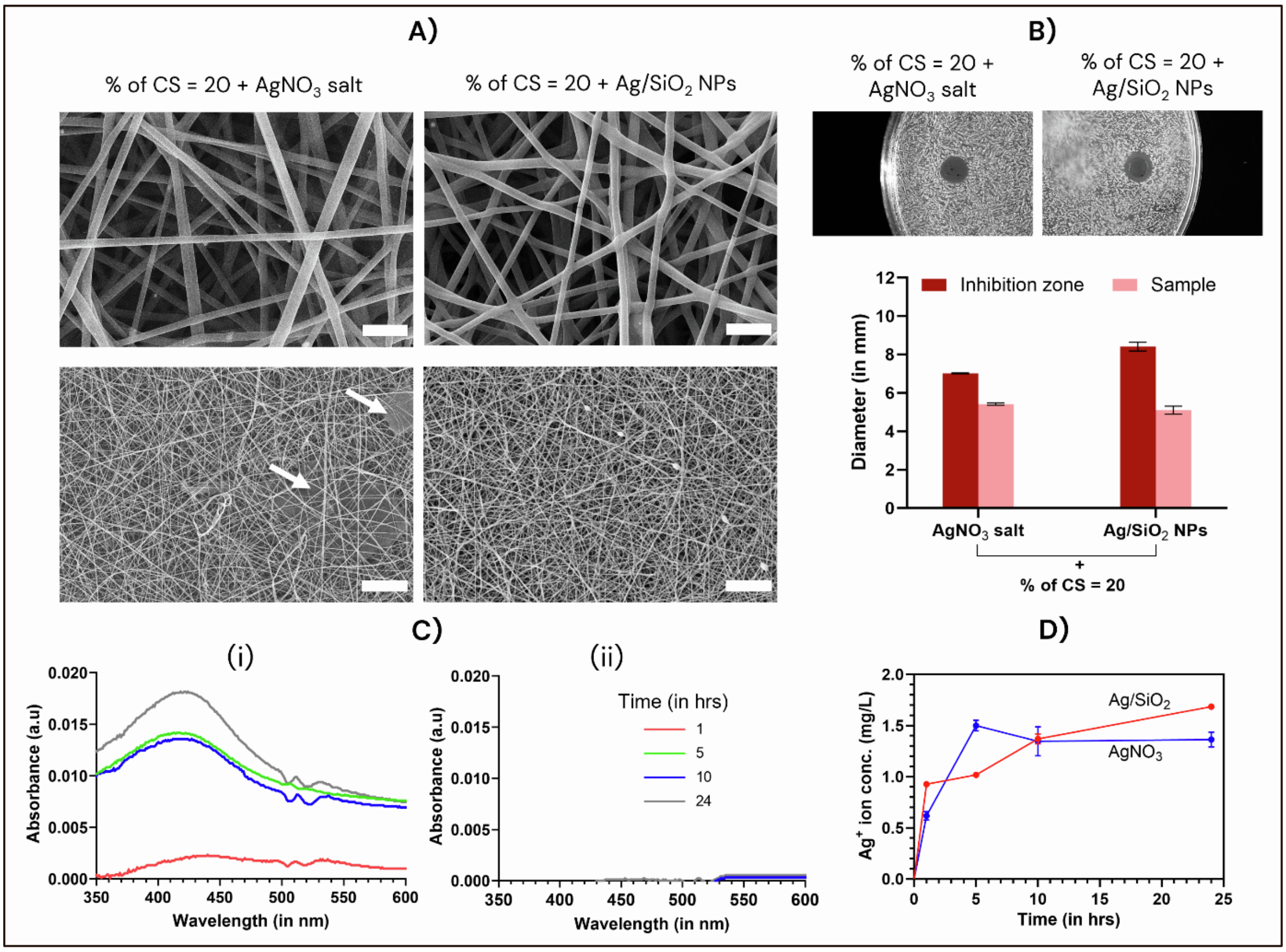

Figure S12: Comparing electrospun membrane containing 20% CS and $AgNO_3$ salt vs. Ag/SiO_2 NPs. A) SEM images of the electrospun membranes. Scale bar= 1 µm (top row), 10 µm (bottom row). White arrows denote the large areas of electrosprayed droplets indicating that $AgNO_3$ salt is less conducive to electrospinning process. B) Disk diffusion method showing the inhibition zone for MRSA after incubation with the membranes for 18 hrs. Data represented as mean ± SEM (n=3). C) Absorbance in the UV-visible range for the membranes containing (i) $AgNO_3$ salt, and (ii) Ag/SiO_2 NPs after immersion in de-ionized water for various intervals of time. In the membrane with $AgNO_3$ salt, the peak at 420 nm corresponds to the surface plasmon resonance of silver nanoparticles and is an indication of their release from the membrane. D) Release profile of Ag^+ from the membranes in de-ionized water at ambient temperature. Data represented as mean ± SD. (N=3)

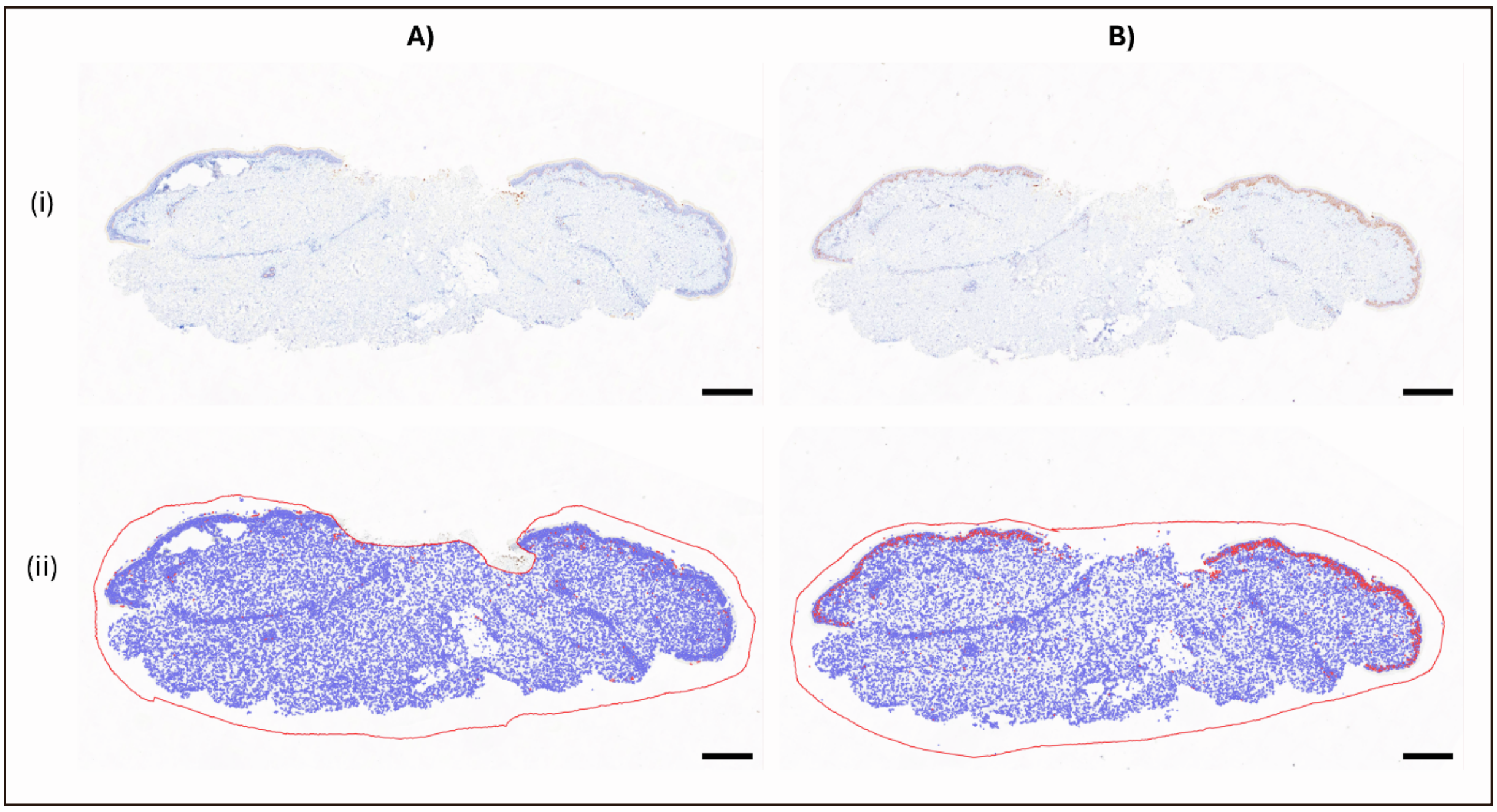

Figure S13: Quantification of cells from immunohistochemistry images (IHC) using QuPath software. The representative images are from tissues incubated with electrospun membranes containing 0% CS and Ag/SiO_2 NPs. The antigens tested using IHC were: A) cleaved caspase-3, indicative of cell apoptosis, and B) PCNA, indicative of cell proliferation. Initially, cell vectors were estimated from raw images (i) followed by demarcation of the region to be processed (red line) in (ii). In sample (A), the Ag/SiO_2 NPs released on the wound surface from the membranes also absorbed the stains, resulting in false positives. To mitigate this, the NPs were excluded during the demarcation process in (A). The total number of cells was detected from the optical density of Hematoxylin, indicated by blue in (ii). The percentage of these cells positive for the antigens was then determined by the optical density of DAB stain, indicated by red in (ii). Scale bar= 500 µm.

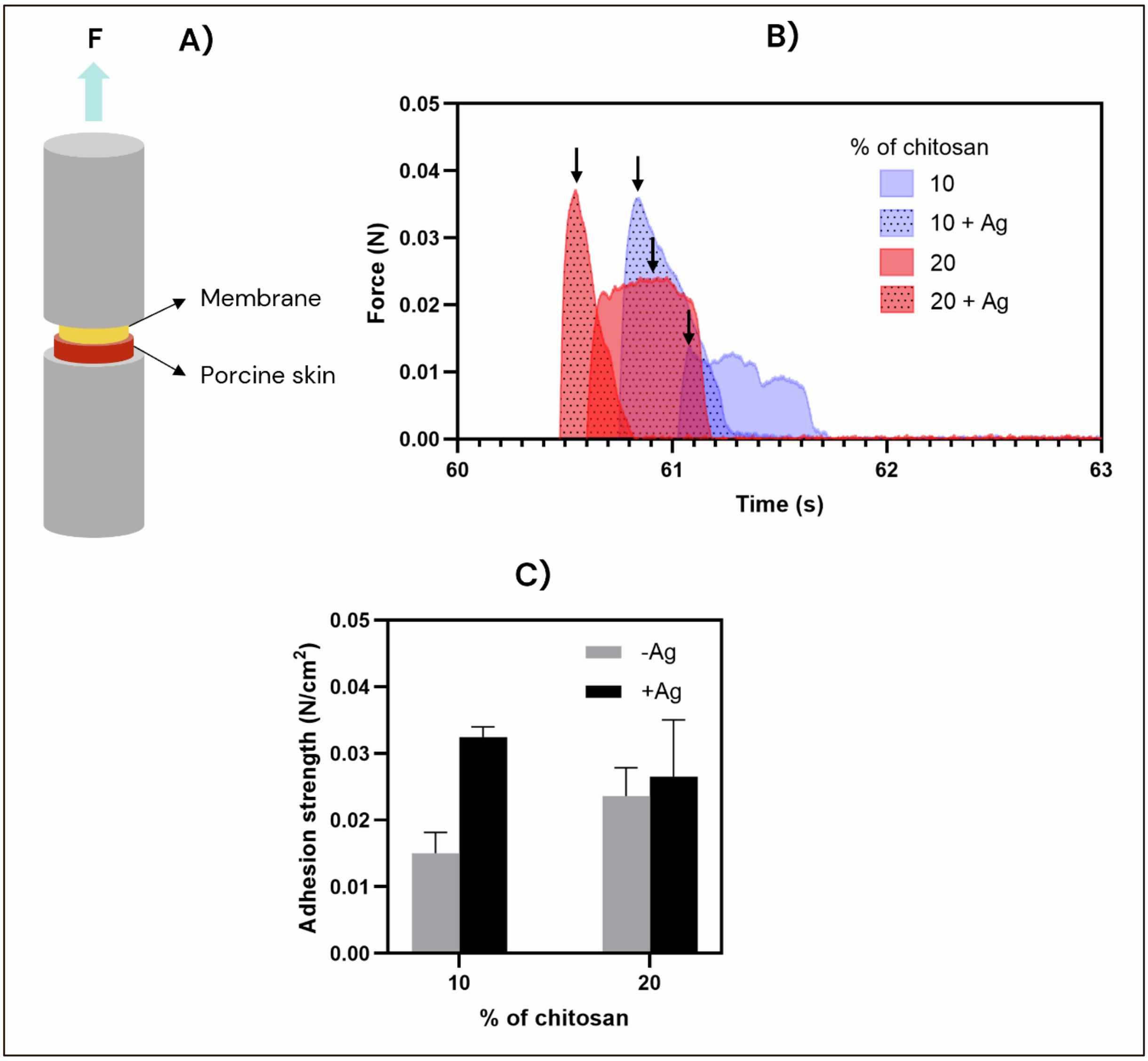

Figure S14: Estimating the tensile adhesion strength of the electrospun membranes on porcine skin using texture analyzer. Thawed porcine skin was incubated at 37 °C for 2 hours before the test to simulate physiological conditions. The electrospun membranes contained 10, 20% of chitosan with and without Ag/SiO_2 NPs. A) Schematic showing the experimental set-up. A 15 mm diameter cylindrical probe was used to apply the membrane on the skin with a force of 0.1 N for 60 s after which the membrane was detached at a speed of 0.5 mm/s. B) Force-time curve during removal of membrane from the skin. Black arrows indicate the maximum force required to detach the membrane, representing the adhesion strength, as shown in C). Data represented as mean ± SEM (N=3).

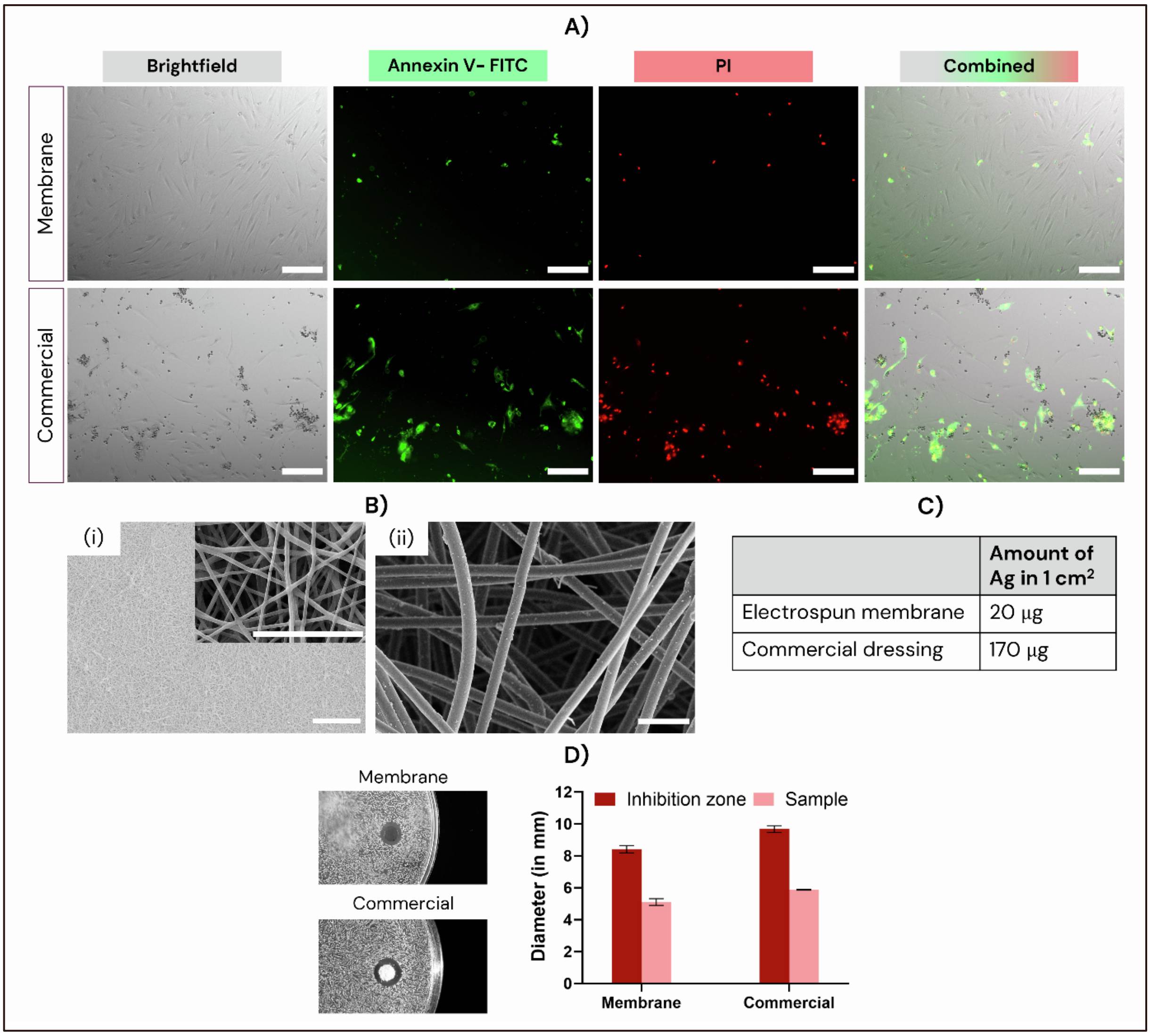

	Amount of Ag in 1 cm^2
Electrospun membrane	20 µg
Commercial dressing	170 µg

Figure S15: Comparing electrospun membrane containing 20% CS and Ag/SiO_2 NPs with commercial silver-based dressing. A) Fluorescence microscopy images of fibroblasts, 72 hours after incubation with extracts of the samples. Brightfield images for the electrospun membrane show the characteristic elongated spindle morphology of fibroblasts while significant number of floating dead cells are seen for the commercial dressing. Cells undergoing apoptosis are stained green by Annexin V-FITC while dead cells are stained red by PI, thereby showing the cytotoxic effects of the commercial dressing. Scale bar= 200 µm. B) SEM images of (i) electrospun membranes, and (ii) Commercial dressing. Scale bar= 50 µm, 5 µm (inset). C) Table showing amount of silver present in the samples. D) Disk diffusion method showing the inhibition zone for MRSA after incubation with the samples for 18 hrs. Data represented as mean ± SEM (N=3).

Table S1: Comparison with literature on silver nanoparticles (Ag NPs) in electrospun fibers for antibacterial activity against S. aureus. #denotes that the initial diameter of the sample for disk diffusion study was not provided and was assumed to be the standard 6 mm. *denotes that the inhibition zone was measured after 3 days of incubation instead of the standard 18 hours, leading to a higher observed antibacterial activity. Abbreviations used here are- MADO: poly(dopamine methacrylamide-co-methyl methacrylate), PCL: polycaprolactone, PVA: polyvinyl alcohol, PLA: polylactic acid, PEO: poly ethylene oxide, CA: cellulose acetate, PLLA: poly L-lactic acid, PVP: polyvinylpyrrolidone

Ref.	Ag NPs		Polymer for electro-spinning	Incorporation of silver	Target microbe	*In vitro* antibacterial efficiency			Bio-compatibility
	Preparation	Size (nm)				Disk diffusion	OD_{600}	CFU count	
[1]	Reduction on fiber surface by dopamine	18	MADO	Fibers dipped in $AgNO_3$	*S.aureus*	1.3 mm inhibition zone	-	-	Fibroblasts
[2]	UV-irradiation of fibers	8	Silk fibroin	$AgNO_3$ mixed with polymer	*S.aureus*	1.5 mm	-	-	Fibroblasts
[3]	Reduction of $AgNO_3$ by *P.nigram* extract	5-20	PCL	Ag NPs mixed with polymer	*S.aureus*	5.6 mm	-	-	-
[4]	Reduction of $AgNO_3$ by citrate	25-55	Collagen	Ag NPs mixed with polymer	*S.aureus*	6 mm#	-	-	Rat skin
[5]	Reduction of $AgNO_3$ by tannic acid	11-17	PVA	Ag/PVP NPs mixed with polymer	*S.aureus*	-	0.01	-	-

[6]	Reduction of $AgNO_3$ by DMF	20-50	PLA	GO-Ag NPs mixed with polymer	*S.aureus*	3 mm	0.01	-	
[7]	Reduction on fiber surface by mucin	80	Jellyfish biomass, PCL	Fibers dipped in $AgNO_3$	MRSA	-	0.01	-	Fibroblasts
[8]	Reduction of $AgNO_3$ by chitosan	20	PEO+ chitosan	$AgNO_3$ mixed with polymer	*S.aureus*	5 mm	-	-	
[9]	Laser ablation of silver plate	40	CA, PCL	Ag NPs mixed with polymer	*S.aureus*	6 mm*			Osteoblast
[10]	Reduction on fiber surface by dopamine		PLLA	Fibers dipped in dopamine + $AgNO_3$	*S.aureus*	4 mm	-	-	Fibroblasts
[11]	Reduction on fiber surface by chitosan	7	PCL	Fibers dipped in $AgNO_3$ + chitosan	*S.aureus*	-	-	1-log reduction	Osteoblast
[12]	UV-irradiation of fibers	2-5	PVP	$AgNO_3$ mixed with PVP	*S.aureus*	2 mm	-	-	Vero cells
**This work**	**Flame spray pyrolysis of silver acetate**	**2-10**	**PVA, chitosan**	**Ag/SiO_2 NPs mixed with polymer**	**MRSA**	**3.4 mm**	**0.002**	**9-log reduction**	**Human skin Fibroblasts Keratinocytes**

Protocol for generating bivariate histogram from electron tomographs

The bivariate particle frequency histograms in Figure S5 were generated with the following approach. Tomograms were filtered in 2D along the x-y plane using non-local means to enhance the contrast of nanoparticles. These were subsequently segmented using an adaptive thresholding approach. Following segmentation, individual particles were identified through a 3D connected components analysis, with a minimum size threshold of three voxels to classify an object as a nanoparticle. Volumetric properties, including minimum inscribed sphere diameter and three-dimensional centroids of the segmented particles, were quantified using the regionprops function in the Matlab Image Processing Toolbox. The minimum inscribed sphere diameter is used for the particle diameter histograms while the centroids were used to determine particle spatial distributions.

To estimate the fiber axis, the combined volume of the fiber and associated nanoparticles was first segmented from the background. Singular value decomposition (SVD) was then applied to the voxel coordinates of this segmented volume, and the principal axis corresponding to the greatest variance was selected as the fiber axis.

Fiber thickness was quantified by casting azimuthally discretized, orthogonal rays from the estimated fiber axis. The lengths of these rays were measured up to the intersection with the boundary defined by the segmentation threshold. These radial vectors were individually normalized to unit length. To assess the spatial distribution of nanoparticles, the normalized radial distances and axial positions of the centroids were binned, and the frequency of particles within each bin was used to construct binary histograms representing their spatial organization relative to the fiber.

References

[1] A. GhavamiNejad, A. Rajan Unnithan, A. Ramachandra Kurup Sasikala, et al., *ACS Appl Mater Interfaces* **2015**, *7*, 12176.

[2] S. Calamak, E. A. Aksoy, N. Ertas, C. Erdogdu, M. Sagıroglu, K. Ulubayram, *Eur Polym J* **2015**, *67*, 99.

[3] R. Augustine, N. Kalarikkal, S. Thomas, *Appl Nanosci* **2016**, *6*, 337.

[4] G. Rath, T. Hussain, G. Chauhan, T. Garg, A. K. Goyal, *J Drug Target* **2016**, *24*, 520.

[5] Z. Zhang, Y. Wu, Z. Wang, X. Zou, Y. Zhao, L. Sun, *Materials Science and Engineering: C* **2016**, *69*, 462.

[6] C. Liu, J. Shen, K. W. K. Yeung, S. C. Tjong, *ACS Biomater Sci Eng* **2017**, *3*, 471.

[7] R. Nudelman, H. Alhmoud, B. Delalat, et al., *Adv Funct Mater* **2019**, *29*, 1902783.

[8] J. Shao, B. Wang, J. Li, J. A. Jansen, X. F. Walboomers, F. Yang, *Materials Science and Engineering: C* **2019**, *98*, 1053.

[9] M. K. Ahmed, A. A. Menazea, A. M. Abdelghany, *Int J Biol Macromol* **2020**, *155*, 636.

[10] J. Wang, L. Zhan, X. Zhang, R. Wu, L. Liao, J. Wei, *Front Pharmacol* **2020**, *11*.

[11] D. Porrelli, M. Mardirossian, L. Musciacchio, M. Pacor, F. Berton, M. Crosera, G. Turco, *ACS Appl Mater Interfaces* **2021**, *13*, 17255.

[12] A. S. Pádua, R. M. Rosa, T. Vieira, C. Henriques, J. C. Silva, *J Drug Deliv Sci Technol* **2024**, *101*, 106303.
